# The Efficacy of Experiential Dynamic Therapies: A 10‐Year Systematic Review and Meta‐Analysis Update

**DOI:** 10.1002/cpp.70086

**Published:** 2025-05-23

**Authors:** Peter Lilliengren, Jakob Mechler, Karin Lindqvist, Daniel Maroti, Robert Johansson

**Affiliations:** ^1^ Department of Psychology Stockholm University Stockholm Sweden; ^2^ Department of Psychology Uppsala University Uppsala Sweden

**Keywords:** affect‐focused, emotion regulation, experiential, meta‐analysis, psychodynamic, transdiagnostic

## Abstract

There is a growing interest in clinical interventions targeting emotion regulation difficulties across mental health conditions. Experiential dynamic therapies (EDTs) are transdiagnostic, affect‐focused, short‐term psychodynamic therapy models that emphasize in‐session emotional processing. This review provides a 10‐year update on the efficacy of EDTs for mood, anxiety, personality and somatic symptom disorders in adults and children/adolescents. A comprehensive search identified 57 randomized controlled trials (*n* = 4330) conducted in Western (*k* = 38; *n* = 3178) and non‐Western countries (*k* = 19; *n* = 1152) between 1978 and 2024. Random‐effects meta‐analyses on primary outcomes indicated large, significant effects for EDTs compared to inactive controls at post‐treatment (Hedge's *g* = −0.96; *k* = 41) and follow‐up (*g* = −1.11; *k* = 20). Compared to active controls, effects were small and non‐significant post‐treatment (*g* = −0.17; *k* = 27) but became significant at follow‐up (*g* = −0.40; *k* = 19), suggesting a potential modest long‐term advantage of EDTs. Despite substantial heterogeneity (*I* ^*2*^ > 75%), results remained robust in sensitivity analyses. Moderator analyses revealed few significant findings, indicating relative consistency across diagnostic groups, treatment formats and active comparators. Non‐Western and lower quality studies reported larger effects compared to inactive, but not active, controls. While cautious interpretation is warranted due to unexplained heterogeneity, findings support EDTs as efficacious transdiagnostic interventions for emotional disorders, with sustained benefits over time. Future research should prioritize large‐scale, methodologically rigorous trials that explore mechanisms of change, optimize treatment delivery and identify moderators of long‐term outcomes.

**Summary**

- Experiential Dynamic Therapies (EDTs) show large and sustained effects compared to inactive controls across common mental health conditions associated with maladaptive emotion regulation.
- The short‐term effects of EDTs are similar to other active treatments, but EDTs may have modest advantages in the long run.
- While results suggest EDTs are effective overall, cautious interpretation is warranted due to substantial differences between studies.

## 1 Introduction

In recent years, multiple lines of research have identified maladaptive emotion regulation as a core underlying process contributing to a wide range of mental health conditions, including mood, anxiety, personality, substance use and somatic symptom disorders (e.g., Aldao et al. 2016; Carmassi et al. 2022; Schnabel et al. 2022; Sheppes et al. 2015; Sloan et al. 2017). Research further suggests dysfunctional emotion regulation may be a mediator between adverse childhood experiences and psychiatric disorders (e.g., Compas et al. 2017; Malik et al. 2015; Miu et al. 2022), emphasizing its potential role for understanding developmental psychopathology (Cavicchioli et al. 2023). This growing understanding has fuelled interest in transdiagnostic psychotherapeutic interventions that specifically target emotion regulation (e.g., Barlow et al. 2020; Berking and Lukas 2015; Renna et al. 2017). Since such interventions address a common mental health vulnerability and focus on a core mechanism of change across different conditions, they provide a unified approach to managing the issue of psychiatric comorbidity, which is highly prevalent in clinical practice (e.g., Kessler et al. 2005). This may optimize psychotherapy training, streamline treatment, and facilitate the dissemination of evidence‐based interventions to clinical settings (Leichsenring and Steinert 2017; Schaeuffele et al. 2021).

One increasingly popular group of therapy models that emphasize in‐session emotional processing as a transdiagnostic change agent is experiential dynamic therapies (EDTs; Osimo and Stein 2012). Rooted in psychodynamic terminology and understanding of psychopathology, these models view psychological symptoms and/or problematic interpersonal patterns as the consequence of an individual's (conscious and/or unconscious) attempts at regulating intense, conflicted emotions, typically associated with adverse experiences in key attachment relationships during childhood (Abbass 2015; Frederickson 2013, 2020; Fosha et al. 2009). When such conflicted emotions are activated later in life, the individual may experience high levels of anxiety and/or resort to maladaptive, implicit, emotion‐regulating mechanisms (i.e., ‘defences’ in psychodynamic terms; e.g., Grecucci et al. 2020; Rice and Hoffman 2014), leading to relational problems and/or symptom formation.

**FIGURE 1:**
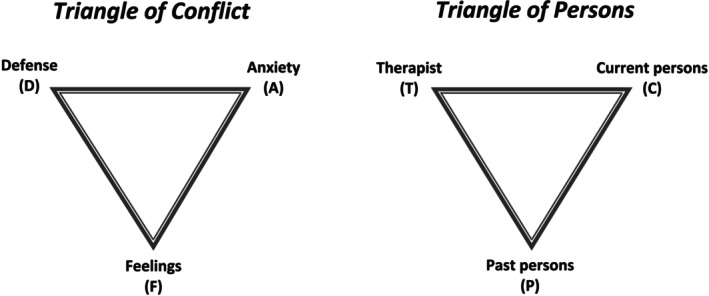
Adopted from Malan (1979). The two triangles illustrate how defences (D) and anxieties (A) block the experience and expression of true feelings (F) and how such patterns began with past persons (P), are maintained with current persons (C) and may be enacted with the therapist (T).

In EDTs, the therapist's main task is to address maladaptive emotion regulation by helping patients identify, experience and integrate conflicted emotions contributing to symptoms and/or other presenting problems. Malan's (1979) two well‐known triangular schemes, i.e., the Triangle of Conflict and the Triangle of Persons (see Figure 1), are used as a framework for conceptualizing the dynamics contributing to and/or maintaining patient difficulties, as well as for the moment‐to‐moment assessment of the patient's in‐session responses and the therapist's corresponding choice of interventions. The overarching aim is to help the patient see and let go of maladaptive defences, regulate excessive anxiety as needed, in order to viscerally experience and process the underlying conflicted emotions. While various EDT models have evolved over time, all share an emphasis on in‐session emotional processing and use the triangles as a foundation for understanding symptom and treatment dynamics.

Several lines of research support the basic treatment principles underlying EDTs. First, patients' in‐session emotional awareness, experience and expression has been linked to outcome across disorders and treatment contexts (e.g., Fisher et al. 2016; Friederich et al. 2017; Keefe et al. 2019; Maroti et al. 2022; Peluso and Freund 2018; Sønderland et al. 2023), supporting its role as a transdiagnostic change process. Consistent with EDTs' emphasis on emotions, a recent meta‐analysis by Diener et al. (2025) found a significant positive relationship between therapists' focus on emotion and patient outcomes in psychodynamic therapies. Additionally, active and skilful work on defences and resistance—a core component of EDTs—has been linked with improved therapeutic alliance (Despland et al. 2001; Gerostathos et al. 2014), greater emotional intensity in sessions (McCullough and Magill 2009; Town et al. 2012) and enhanced long‐term outcomes (Perry and Bond 2017).

EDTs have been developed, practiced and researched since the 1960s and about 10 years ago, our group conducted a first meta‐analysis of randomized controlled trials (RCTs) in this specific area (Lilliengren et al. 2016). The review included a total of 28 studies published up until 2014 and results indicated moderate effects in favour of EDTs compared to inactive controls at both post‐treatment and follow‐up across various outcome domains. We found no significant differences with other active treatments at post‐treatment, but EDTs significantly outperformed manualized supportive therapies at follow‐up. Subgroup analyses based on pre‐post effect sizes suggested EDTs may be most effective when delivered in individual format and in depressive conditions.

However, the review was limited by the relatively low number of included studies and given the general growth in controlled outcome research in psychodynamic therapies over the last decade (Lilliengren 2023), we believe an update is timely. Further, our first review had several methodological shortcomings we aim to address with the present study. First, given the increased interest in using EDTs with children and adolescents (e.g., Derdikman Eiron 2021; Mechler, Lindqvist, Philips, et al. 2024), as well as in somatic symptom disorders and related conditions (e.g., Frisch and Gündel 2023), we want to expand our inclusion criteria to these populations. Secondly, for the previous review, we extracted study data on all measures of psychopathology and combined both general and specific symptom measures into one domain labelled ‘general psychiatric symptoms’. While this procedure was judicious given the aims of the initial review, it may have obscured treatment effects in targeted conditions. This time, therefore, we will follow recommendations in the field (e.g., Higgins et al. 2021; Moher et al. 2009) and focus on the primary outcome of each study. This will provide a clearer view of the effects of EDTs in specific diagnostic areas and enable more direct comparisons with other reviews. Lastly, due to the limited number of included studies in our prior review, we were only able to conduct moderator analyses on uncontrolled pre‐post effect sizes. Since pre‐post effects are influenced by extraneous factors such as regression to the mean and natural recovery, they may overestimate treatment effects (Cuijpers et al. 2017). Therefore, this review will focus exclusively on between‐group effect sizes.

In summary, our aim with this review is to synthesize findings from RCTs evaluating the efficacy of EDTs for common mental health conditions in adult and child/adolescent populations. We will assess the effects of EDTs on primary outcomes at post‐treatment and follow‐up in comparison to both inactive (e.g., waitlist) and active (e.g., CBT, medication) control conditions. Additionally, we will explore potential moderators of treatment effects, including study quality, to identify factors that may be related to outcomes. By integrating recent findings and incorporating the methodological improvements described above, this study will provide a comprehensive, up‐to‐date evaluation of EDTs, advancing our understanding of their potential as transdiagnostic interventions for mental health conditions associated with maladaptive emotion regulation.

## 2 Methods

This review was pre‐registered in PROSPERO (CRD42024558834) and the reporting follows recommendations outlined in the Preferred Reporting Items for Systematic Reviews and Meta‐analysis (PRISMA) guidelines (Page et al. 2021).

### 2.1 Identification and Selection of Studies

Searches were part of an ongoing project to compile all RCTs ever published on psychodynamic treatments and interventions (i.e., Lilliengren 2017, 2023). Since 2014, the first author has performed recurrent manual searches four to five times per year in major databases, including PubMed, PsycINFO, Cochrane Library and Google Scholar, to identify RCTs and meta‐analyses of psychodynamic therapies. No restrictions were applied regarding publication date, type or original language. All retrieved studies and meta‐analyses have been examined for references to previously undetected trials. Additionally, the first author has had regular contact with leading psychodynamic researchers to inquire about lesser‐known or unpublished studies. The present review includes results of searches that have been conducted until 30 December 2024 (see Supplement for specific terms used in latest search).

The primary inclusion criterion was that the intervention under study fit our definition of EDT, which is any treatment model directly based on or derived from the works of Malan and/or Davanloo. Established EDT‐models include Malan's Brief Dynamic Therapy (BDT; Malan 1979), Davanloo's Intensive Short‐Term Dynamic Psychotherapy (ISTDP; Davanloo 2000), Fosha's Accelerated Experiential‐Dynamic Psychotherapy (AEDP; Fosha 2000), Osimo's Experiential Short‐Term Dynamic Psychotherapy (E‐STDP; Osimo 2003), McCullough's Affect Phobia Therapy (APT; McCullough et al. 2003) and Schubiner's and Lumley's Emotional Awareness and Expression Therapy (EAET; Lumley and Schubiner 2019). To determine eligibility, two researchers (P. L. and R. J.) screened all identified psychodynamic RCTs (*k* = 362) for explicit references to Malan or Davanloo or any of their known successors in the treatment descriptions. When necessary, treatment manuals, books or articles referenced were also consulted to confirm classification. Studies citing Malan and/or Davanloo alongside other theorists were retained, as were integrative EDT approaches (e.g., Orvati Aziz et al. 2020).

Studies that met the overall EDT criterion were then read in detail to determine additional inclusion criteria. Each study had to (1) target a clinical population diagnosed using a formal system (e.g., DSM, ICD) or other established procedure for the targeted condition (e.g., a disorder specific interview or defined cut‐off on a specific measure), (2) deliver EDT as individual therapy, group therapy or internet‐based treatment, (3) use randomized allocation of participants and (4) report sufficient data for the calculation of effect sizes on a primary outcome. To evaluate these criteria, as well as assess study quality (see below), only studies published full‐text in English were considered. Studies were further excluded if they (a) evaluated single‐session interventions (as EDTs typically involve eight to 40+ sessions in clinical practice) or (b) were parametric studies comparing variations of EDTs (e.g., long‐term versus short‐term), unless a separate control condition was present for comparison (e.g., Mechler, Lindqvist, Magnusson, et al. 2024; Rahmani, Abbass, Hemmati, Ghaffari, et al. 2020; Rahmani, Abbass, Hemmati, Mirghaed, et al. 2020).

Two researchers in the team (P. L. and R. J.) collaboratively assessed the inclusion and exclusion criteria and differences in opinion were discussed until consensus was reached. Next, one researcher (P. L.) extracted data for effect sizes, study characteristics and moderator variables (except study quality, see below). To ensure coding integrity, a second researcher (R. J.) independently coded a random subset of studies (*k* = 12, 21%). Interrater reliability was excellent across extracted study variables, with intraclass correlation coefficients (ICC [2,1]) ranging from 0.89 to 0.92 and Cohen's kappa (κ) ranging from 0.77 to 1.00, indicating a high degree of coding consistency.

### 2.2 Meta‐Analytic Procedures

Data from post‐ and follow‐up assessments (when available) on each study's primary outcome were extracted to compute effect sizes. If multiple follow‐ups were reported, we used the final assessment, and data from separate follow‐up publications were also included when available. When multiple measures were designated as primary, they were combined and assumed to be perfectly correlated (*r* = 1), as this is the more conservative approach when the true correlation is unknown (Borenstein et al. 2009). If no primary outcome was specified, we selected the measure most relevant to the condition treated. One study (Johansson et al. 2013) included two independent samples—one with primary mood disorders and one with primary anxiety disorders—reporting outcomes on anxiety and depression measures separately. These samples were treated as independent studies, with separate effect sizes calculated for each sample.

For our primary analyses, comparison conditions were categorized as either ‘Inactive’ or ‘Active’ controls. Inactive controls included no treatment, waitlist, treatment‐as‐usual (TAU) and limited treatment controls (e.g., unstructured support groups, minimal contact, clinical monitoring). While some of these controls may have effects of their own (e.g., Munder et al. 2022), they were typically described as non‐standardized, lower‐intensive interventions by the study investigators and categorizing them as inactive is arguably the most conservative approach. Additionally, in studies evaluating the added effect of EDT to medication, the medication‐alone control condition was categorized as an inactive control, as the combined treatment was expected to yield EDT‐specific effects (Driessen et al. 2020).

Active controls included other bona‐fide psychotherapies (Wampold et al. 2002) of equal duration to EDTs, as well as active medication protocols when compared directly to EDTs without medication. If a study compared EDT with two active treatments (e.g., Dare et al. 2001), the active comparators were combined to ensure only one effect size from each study. One study (Lumley et al. 2017) used two control groups defined as active treatments by the study authors (CBT and fibromyalgia education group). However, we decided to code the educational group as a limited treatment control (and, hence, inactive in our analysis) since patients had less time with a trained therapist. In another study (Rahmani, Abbass, Hemmati, Mirghaed, et al. 2020), four EDT variations were combined to prevent double‐counting the same waitlist control. In Mechler, Lindqvist, Magnusson, et al. (2024) and Mechler, Lindqvist, Philips, et al. (2024), only the therapist‐guided version of internet‐based EDT was included, as it better represents real‐world clinical practice. For studies that involved both an inactive and an active control group, effect sizes were calculated and analysed separately. Thus, each study could only contribute one effect size per comparison. The effects of EDTs in comparison with specific categories of inactive and active controls were explored in subgroup analyses (see further below).

Between‐group effect sizes (Cohen's *d*) were calculated at post‐treatment and follow‐up. In line with recommendations to minimize bias due to missing data (Higgins et al. 2021), estimates derived from intent‐to‐treat (ITT) analyses using linear mixed models (LMMs) were used when reported (Feingold 2017, Equation 2). When ITT data was unavailable, effect sizes were computed using the reported *n*, *M* and *SD*s of each group. Data errors were detected in two studies (Chavooshi et al. 2016a, 2016b), where standard errors were mistakenly reported as standard deviations. After contacting the authors, we corrected these values accordingly. For dichotomous outcomes, risk ratio (RR) were converted to Cohen's *d* using the logit method (Borenstein et al. 2009). All effect sizes were standardized so that negative values indicate a benefit of EDT over the comparison condition (i.e., lower scores on the primary outcome). To address potential bias from small sample sizes, all effect sizes were transformed to Hedge's *g* before the main analyses (Borenstein et al. 2009). Following conventions in the field (Cohen 1988), effect sizes were categorized as small (≥ 0.20), medium (≥ 0.50) or large (≥ 0.80).

We also examined dropout rates between EDTs and active control conditions. Data was extracted from study flow‐charts or tables indicating the number of participants remaining at post‐treatment. Results were synthesized using log risk ratios (log RR), which create a symmetric metric around zero and simplifies interpretation: positive values indicate a higher risk of dropout in EDT, while negative values indicate a lower risk (Higgins et al. 2021).

All meta‐analytic calculations were performed using IBM SPSS Statistics, version 29.0.2.0 (IBM Corp. 2023). A random‐effects model was applied since we assumed that true effects would vary across studies given the broad inclusion criteria, enhancing generalizability (Borenstein et al. 2009). The Sidik–Jonkman method (Sidik and Jonkman 2002) was used to estimate between‐study variance, offering improved stability and robustness compared to traditional approaches. Additionally, the Knapp–Hartung adjustment for standard errors (Knapp and Hartung 2003) was applied, as it accounts for uncertainty in variance estimates, producing more reliable confidence intervals. These adjustments are particularly beneficial when including small studies or in the presence of substantial between‐study heterogeneity (IntHout et al. 2014).

Heterogeneity was assessed using *Q*‐statistics and the *I* ^*2*^‐estimate. The *Q*‐statistic tests whether the observed variability in effect sizes is greater than expected by chance alone, with a significant result suggesting the presence of heterogeneity. The *I* ^*2*^‐estimate complements the *Q*‐statistic by quantifying the proportion of total variation in effect sizes attributable to true heterogeneity rather than sampling error. Expressed as a percentage, *I* ^*2*^‐values provide an intuitive measure: 0% indicates no heterogeneity, ≥ 25% represents low heterogeneity, ≥ 50% indicates moderate heterogeneity and ≥ 75% signifies a high level of heterogeneity (Higgins et al. 2003).

To assess the robustness of our pooled estimates, we inspected forest‐plots for potential outliers (defined as study 95% CI fully outside the 95% CI of the pooled effect on either side) and re‐ran analyses with outliers removed. We also examined funnel‐plots and computed Egger's regression‐based test for funnel‐plot asymmetry, which may indicate influence of publication bias and/or small samples on heterogeneity. In addition, we applied Duval and Tweedie's (2000) trim‐and‐fill method, which yields an adjusted estimate of the pooled mean effect size and its 95%‐confidence intervals, after publication bias has been considered. The random‐effects model with Knapp–Hartung adjusted standard errors was applied in these procedures as well. Results of sensitivity analyses are reported when the effect size estimate notably altered (*g* ≥ ±0.15), heterogeneity significantly changed (*I* ^*2*^ change ≥ ± 15%) and/or statistical significance changed (e.g., *p* > 0.05).

### 2.3 Moderator Analyses

To explore heterogeneity and the potential impact of study‐level moderators, we conducted subgroup analyses of categorical variables (e.g., type of control) and meta‐regression models for continuous moderators (e.g., year of publication). All analyses were conducted separately for EDTs compared to inactive and active control conditions at post‐treatment and follow‐up.

For subgroup analyses, inactive controls were categorized as ‘Waitlist/no treatment’, ‘TAU’, ‘Limited treatment’ or ‘Medication‐alone’ (i.e., in add‐on studies evaluating EDT combined with medication). Active psychotherapies where coded as ‘CBT’ (for interventions based on cognitive and/or behavioural theories), ‘Other psychotherapy’ (e.g., family therapy), ‘Manualized supportive therapy’ (when the intervention was of equal duration and guided by a manual) and ‘Medication’ (when EDT was directly compared to an active medication protocol). Studies were also grouped by their targeted condition, i.e., ‘Mood disorders’, ‘Anxiety disorders’, ‘Personality disorders’ and ‘Somatic symptom disorders’. Study samples that did not fit with these categories or encompassed participants with several primary conditions were categorized as ‘Mixed/other’. Targeted study age groups were coded ‘Adults’ (i.e., > 18 years) or ‘Children/adolescents’ (< 18 years) and treatment formats were coded ‘Individual therapy’, ‘Group therapy’ or ‘internet‐based treatment’. Studies were also coded ‘Western’ if conducted in Europe or North America and ‘non‐Western’ when conducted elsewhere in the world.

Random‐effects models using the Sidik–Jonkman method was employed for subgroup analyses due to its stability in estimating between‐study variance, even in smaller subgroups (Sidik and Jonkman 2002). However, the Knapp–Hartung adjustment was not applied as it may produce overly conservative confidence intervals when subgroups contain a limited number of studies (Röver et al. 2015). Given the common problem of low power in subgroup analyses (Cuijpers et al. 2021), overly conservative intervals may underestimate meaningful differences, as well as obscure potential outliers, limiting interpretability. To maintain statistical stability and minimize spurious findings, only subgroups containing at least three studies were considered (Borenstein et al. 2009).

To test for subgroup differences, we used the ANOVA based method where the between‐group *Q*‐statistic (*Q*_*between*_) is equivalent to the *F*‐statistic (Borenstein et al. 2009). A significant Q_between_ indicates variation between subgroups but does not specify which pairs differ. When significant, post hoc *Q*‐tests were conducted to examine pairwise subgroup differences.

For meta‐regression analyses, we used random‐effects models with Sidik–Jonkman and Knapp–Hartung adjustments to account for both within‐ and between‐study variability. Weighted regression was applied, assigning greater weight to larger sample studies due to their more precise effect size estimates (Borenstein et al. 2009). The continuous moderator served as the predictor, while effect sizes for EDT versus inactive and active controls were used as the dependent variables in separate analyses.

### 2.4 Study Quality

Study quality was assessed using the Randomized Controlled Trial of Psychotherapy Quality Rating Scale (RCT‐PQRS; Kocsis et al. 2010). This scale was specifically developed for assessing psychotherapy trials and involves 24 items referring to central design elements, such as the description of inclusion criteria, diagnostic procedures, delivery of treatment, random assignment, outcome measurement and data analysis, etc. Each item is rated from 0 (poor description, execution or justification of a design element) to 2 (well described, executed and justified design element). Thus, the total score of the scale ranges from 0 to 48 and the suggested cut‐off score for adequate study quality is 24 (Gerber et al. 2011). The instrument has been used in several meta‐analyses (e.g., Keefe et al. 2020; Leichsenring et al. 2023; Thoma et al. 2012) reporting excellent internal consistency and inter‐rater reliability.

For this study, three members of the research team (J. M., K. L. and D. M.) rated the included studies with the RCT‐PQRS. All three raters are PhD‐level researchers with experience in conducting RCTs. Total score inter‐rater reliability was excellent (ICC [2, 1] = 0.76; Cicchetti 1994) based on a random sample of 12 studies (21%) rated by all three raters independently. The remaining studies were divided equally between the raters, making sure they did not assess studies in which they were among the authors themselves. The impact of study quality on the effects of EDTs in comparison with inactive and active controls was assessed with the same meta‐regression procedures described above and is reported under a separate heading in the Results section below.

## 3 Results

### 3.1 Description of Included Studies

**FIGURE 2:**
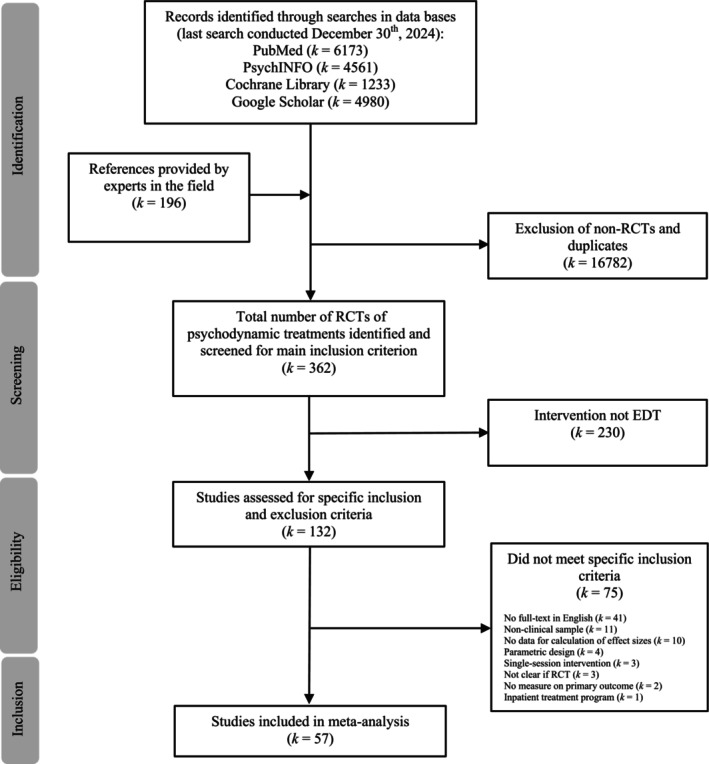
PRISMA flow‐chart of the study selection process.

The study selection process is presented in Figure 2. From the pool of 362 psychodynamic RCTs identified in searches, 132 studies met the primary inclusion criteria. After detailed assessment of the specific inclusion criteria, 57 studies (58 independent samples) were included in the meta‐analysis. Full lists of included and excluded studies, along with a table summarizing the study characteristics, are provided in the Supplement.

The included studies were published between 1978 and 2024, encompassing 4330 participants with an average sample size of *n* = 76 (*SD* = 63, range 16–341, Mdn = 57). Two‐thirds of the studies were conducted in Western countries (*k* = 38; *n* = 3178) and one‐third in non‐Western (*k* = 19; *n* = 1152). Fifty‐one studies involved adult participants and six studies involved children and adolescents. Studies targeted mood disorders (*k* = 14), anxiety disorders (*k* = 14), personality disorders (*k* = 6) and somatic symptom disorders (*k* = 14). Other samples involved mixed conditions (*k* = 5), eating disorders (*k* = 2), oppositional defiant disorder (*k* = 2) and unresolved grief (*k* = 1). Regarding treatment format, most studies tested EDTs as individual therapy (*k* = 44), followed by group therapy (*k* = 7) and therapist‐supported, internet‐based treatment (*k* = 6). Treatment sessions were typically delivered weekly, with an average of 15.3 sessions (*SD* = 7.7; range 3–40; *k* = 51) across studies. Follow‐up assessments were reported in 36 studies, with an average duration of 8.9 months (*SD* = 9.8; range 2–48, Mdn = 6) from post‐treatment.

### 3.2 EDTs Versus Inactive Controls

The results are presented in Table 1. At post‐treatment, EDTs showed a large, significant effect compared to inactive controls (*g* = −0.96) with substantial heterogeneity (*I* ^*2*^ = 89%). Inspection of the forest plot (see online Supplement for plots) identified seven potential outliers (four lower and three higher than the pooled effect). After removing these, the effect size was reduced but remained large and significant (*g* = −0.81; 95% CI [−0.95, 0.67]; *p* < 0.001; *k* = 34) with notably lower heterogeneity (*Q* = 60.01; *I* ^*2*^ = 54%). Egger's test indicated some funnel‐plot asymmetry (*p* = 0.04), but the trim‐and‐fill procedure did not lead to imputed studies, indicating no strong evidence of publication bias.

**TABLE 1:** Overall effects for EDTs versus inactive and active controls.

	*k*	*g*	95% CI	*t*	*Q*	*I* ^*2*^
Inactive controls						
Post‐treatment	41	−0.96**	[−1.23, −0.69]	−7.20	191.81**	89%
Follow‐up	20	−1.11**	[−1.53, −0.70]	−5.63	119.72**	89%
Active controls						
Post‐treatment	27	−0.17	[−0.36, 0.18]	−1.85	63.38**	75%
Follow‐up	19	−0.40**	[−0.64, −0.16]	−3.50	56.35**	75%

Abbreviations: CI, confidence interval; EDT, experiential dynamic therapy.

** *p* < 0.01.

At follow‐up, EDTs continued to show a large, significant effect (*g* = −1.11) with a substantial level of heterogeneity (*I* ^*2*^ = 89%). Identifying and removing four potential outliers (two lower and two higher of the pooled effect) did not affect the estimate, nor heterogeneity, significantly (*g* = −1.00; 95% CI [−1.40, 0.61]; *p* < 0.001; *k* = 16; *Q* = 51.17; *I* ^*2*^ = 81%). Egger's test was non‐significant (*p* = 0.22), but the trim‐and‐fill procedure imputed two studies, leading to a reduced, yet still large and significant, estimate (*g* = −0.95; CI [−1.42, −0.49]; *p* < 0.001; *k* = 22).

### 3.3 EDTs Versus Active Controls

Table 1 also presents the effect sizes for EDTs compared to active controls. At post‐treatment, the overall effect of EDT was small and non‐significant (*g* = −0.17), with a high level of heterogeneity (*I* ^*2*^ = 75%). Inspection of the forest plot (see Supplement) identified two potential outliers lower than the pooled estimate. Removing these studies reduced the effect size (*g* = −0.09; 95% CI [−0.23, 0.05]; *t* = −1.32; *p* = 0.200; *k* = 25) and lowered heterogeneity (*Q* = 32.93; *I* ^*2*^ = 58%). Egger's test was non‐significant and there was no indication of publication bias.

**FIGURE 3:**
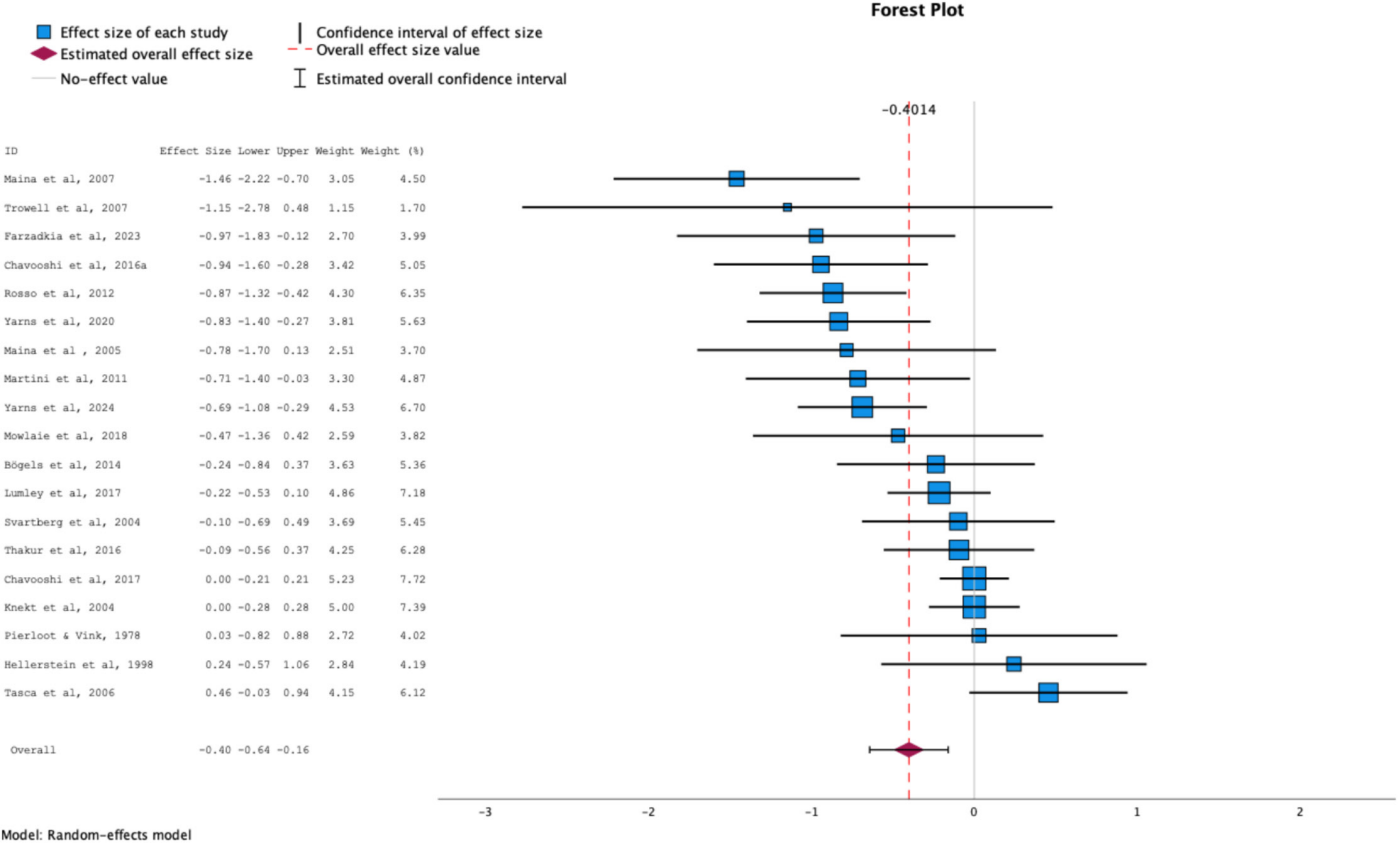
Forest‐plot for EDTs versus active treatments at follow‐up.

At follow‐up, the effect remained small but reached statistical significance (*g* = −0.40), again with a high level of heterogeneity (*I* ^*2*^ = 75%). Inspection of forest plot (see Figure 3) identified one outlier on each side of the pooled estimate. Removing these did not meaningfully alter the effect size (*g* = −0.39; 95% CI [−0.60, −0.19]; *p* = 0.001; *k* = 17), nor significantly reduce heterogeneity (*Q* = 38.37; *I* ^*2*^ = 64%). Egger's test remained non‐significant (see Supplement for funnel plot), but the trim‐and‐fill procedure imputed three studies, reducing the effect size estimate to *g* = −0.31, which was still significant (95% CI [−0.57, −0.06]; *p* < 0.05; *k* = 22).

### 3.4 Dropout Rates

Twenty‐three studies (85%) comparing EDTs with active controls reported data on dropout rates. On average, 13% patients dropped out of EDTs, and the pooled estimate showed no significant difference in dropout risk between EDTs and other active treatments (log RR = −0.08; 95% CI [−0.09, 0.08]; *p* = 0.504; *k* = 23). Heterogeneity was low and non‐significant (*Q* = 13.24; *p* = 0.926; *I* ^*2*^ = 25%), indicating consistency across studies. There was no evidence of significant publication bias or influence from outliers.

### 3.5 Moderator Analyses for EDT Versus Inactive Controls

Results of the subgroup analyses for EDTs versus inactive controls are presented in Table 2. The effect of EDTs differed significantly by type of inactive control at both post‐treatment and follow‐up. At post‐treatment, pairwise post hoc *Q*‐tests showed that the effect against waitlist/no treatment was significantly larger than all other inactive controls (all *p*s < 0.002), while there was no difference among the other controls (*p*s > 0.06). At follow‐up, the effect against waitlist/no treatment remained significantly larger than the other inactive controls (*p*s < 0.001), except for TAU (*p* = 0.97). Egger's test was non‐significant across subgroups. Heterogeneity was high within the waitlist/no treatment subgroup, and removing five outliers at post‐treatment reduced both the effect size (*g* = 0.97; 95% CI [−1.16, −0.77]; *p* < 0.001; *k* = 20) and heterogeneity (*Q* = 34.2; *I* ^*2*^ = 52%). Sensitivity analyses for other subgroups at both time points did not substantially alter estimates or heterogeneity.

**TABLE 2:** Subgroup analyses for EDTs versus inactive controls at post‐treatment and follow‐up.

Subgroups	Post‐treatment					Follow‐up				
	*k*	*g*	95% CI	*I* ^*2*^	*Q*_*between*_	*k*	*g*	95% CI	*I* ^*2*^	*Q*_*between*_
Inactive control conditions					15.75**					15.06**
Waitlist/no treatment	25	−1.27**	[−1.67, −0.87]	89%		10	−1.61**	[−2.21, −1.02]	85%	
TAU	6	−0.68**	[−0.99, −0.36]	46%		3	−1.07*	[−1.96, −0.19]	87%	
Limited treatment	6	−0.53**	[−0.78, −0.28]	34%		3	−0.33*	[−0.64, −0.03]	23%	
Medication‐alone^a^	4	−0.27	[−0.57, 0.03]	33%		4	−0.57*	[−1.51, −0.72]	45%	
Diagnostic categories					4.02					5.90
Mood disorders	8	−1.30**	[−2.24, −0.35]	96%		4	−1.21**	[−2.07, −0.19]	90%	
Anxiety disorders	10	−0.90**	[−1.40, −0.40]	85%		3	−0.90	[−1.89, 0.10]	86%	
Personality disorders	4	−0.55*	[−1.04, −0.07]	55%		—				
Somatic symptom disorders	11	−1.13**	[−1.72, −0.52]	89%		8	−1.30**	[−1.88, −0.72]	89%	
Mixed/other	8	−0.72**	[−0.99, −0.45]	38%		—				
Treatment formats					4.23					
Individual therapy	30	−1.09**	[−1.45, −0.73]	90%		17	−1.26**	[−1.69, −0.82]	87%	
Group therapy	5	−0.56*	[−0.92, −0.19]	64%		—				
Internet‐based treatment	6	−0.79**	[−1.06, −0.52]	47%		—				
Age groups					0.20					
Adults	38	−0.97**	[−1.26, −0.68]	90%		19	−1.12**	[−1.53, −0.71]	89%	
Children/adolescents	3	−0.87**	[−1.21, −0.53]	1%		—				
Country					7.66*					23.74**
Western	25	−0.65**	[−0.81, −0.49]	57%		10	−0.46**	[−0.66, −0.26]	33%	
non‐Western	16	−1.51**	[−2.10, −0.92]	91%		10	−1.81**	[−2.32, −1.30]	77%	

Abbreviations: CI, confidence interval; EDT, experiential dynamic therapy; TAU, treatment‐as‐usual.

^a^ Add‐on studies evaluating the effect of adding EDT to medication vs. medication‐alone.

* *p* < 0.05.

** *p* < 0.01.

There were no significant differences in effect sizes across diagnostic groups at either post‐treatment or follow‐up. All subgroups showed moderate to large, significant effects, with moderate to high levels of heterogeneity. At post‐treatment, removing one outlier notably reduced the effect size for mood disorders (*g* = 0.83; 95% CI [−1.21, −0.45]; *p* < 0.001; *k* = 7; *Q* = 34.2; *I* ^*2*^ = 73%), while sensitivity analyses did not notably change other estimates. At follow‐up, removing one outlier in the somatic symptom disorders subgroup slightly reduced the effect size (*g* = 1.12; 95% CI [−1.63, −0.60]; *p* < 0.001; *k* = 7), but heterogeneity remained high (*Q* = 48.16; *I* ^*2*^ = 86%).

There were no significant differences between treatment formats at post‐treatment. Egger's test indicated funnel plot asymmetry for individual therapy at post‐treatment, and removing five outliers reduced the effect (*g* = 0.90; 95% CI [−1.12, −0.68]; *p* < 0.001; *k* = 25) and heterogeneity (*Q* = 48.16; *I* ^*2*^ = 65%). No indication of publication bias or outliers was detected for group therapy or internet‐based treatments. At follow‐up, only two studies examined group therapy and one study tested internet‐based treatment; hence, subgroup analyses could not be performed.

There was no significant difference between adult and child/adolescent samples at post‐treatment. Only three studies examined EDTs versus inactive controls in children/adolescent populations, but the effect size was large (*g* = 0.87) with no heterogeneity (*Q* = 1.65; *I* ^*2*^ = 1%). Follow‐up analyses were not possible, as only one study examined child/adolescent outcomes.

The effects of EDTs against inactive controls differed between Western and non‐Western studies at both post‐treatment and follow‐up, with significantly larger effects reported in non‐Western studies. However, this finding was confounded by differences in specific control conditions, as 81% of non‐Western studies used waitlist/no treatment control compared to 48% of Western studies (*χ* ^*2*^ = 4.53; *p* = 0.03). After restricting the analysis to waitlist‐controlled studies with outliers removed, differences at post‐treatment were no longer significant (*Q*_between_ = 2.22; *p* = 0.14; non‐Western *g* = −1.11; Western *g* = −0.87), while it remained significant at follow‐up (*Q*_between_ = 15.15; *p* < 0.001; non‐Western *g* = −1.68; Western *g* = −0.42).

Meta‐regression analyses indicated no significant relationship between sample size and effects at either at post‐treatment (*β* = 0.004; 95% CI [−0.002, 0.010]; *p* = 0.155; *k* = 41) or follow‐up (*β* = 0.006; 95% CI [−0.001, 0.014]; *p* = 0.104; *k* = 20). The number of EDT sessions was also not associated with effects at post‐treatment (*β* = 0.001; 95% CI [−0.040, 0.043]; *p* = 0.955; *k* = 35) or follow‐up (*β* = 0.066; 95% CI [−0.159, 0.028]; *p* = 0.156; *k* = 19). The duration of follow‐up was also unrelated to the long‐term effects (*β* = 0.026; 95% CI [−0.012, 0.063]; *p* = 0.165; *k* = 20). However, a significant relationship was found between year of publication and effects at both post‐treatment (*β* = −0.034; 95% CI [−0.057, −0.012]; *p* = 0.004; *k* = 41) and follow‐up (*β* = −0.036; 95% CI [−0.068, −0.005]; *p* = 0.025; *k* = 20), suggesting a slight increase in reported effect sizes over time.

### 3.6 Moderator Analyses for EDTs Versus Active Controls

Table 3 presents the results of our subgroup analyses for EDTs compared to active control conditions. At post‐treatment, there was no evidence of significant differences between EDTs and specific types of active treatments. All effects within subgroups were all small and non‐significant, and while heterogeneity was high for CBT and other psychotherapies, there was no indication of publication bias or significant influence from outliers. At follow‐up, the between subgroup *Q*‐test was also non‐significant, suggesting relative consistency across subgroups, although the specific subgroup estimates varied more. Heterogeneity remained moderate to high within subgroups, but there was no indication of publication bias or significant outlier influence. A forest plot and separate funnel plots for subgroups are provided in the Supplement for this analysis.

**TABLE 3:** Subgroup analyses for EDTs versus active controls at post‐treatment and follow‐up.

Subgroup analyses	Post‐treatment					Follow‐up				
	*k*	*g*	95% CI	*I* ^*2*^	*Q*_*between*_	*k*	*g*	95% CI	*I* ^*2*^	*Q*_*between*_
Active control conditions					1.31					2.59
CBT	11	−0.20	[−0.48, 0.09]	80%		8	−0.20	[−0.50, 0.09]	72%	
Other psychotherapy	8	−0.28	[−0.78, 0.22]	85%		6	−0.44*	[−0.85, −0.03]	60%	
Manualized supportive	5	−0.15	[−0.44, 0.15]	8%		5	−0.74**	[−1.27, −0.20]	66%	
Medication^a^	3	0.04	[−0.35, 0.43]	10%		—				
Diagnostic categories					8.68					18.60**
Mood disorders	7	−0.14	[−0.33, 0.05]	10%		4	−1.01**	[−1.42, −0.61]	73%	
Anxiety disorders	6	−0.03	[−0.45, 0.40]	46%		4	−0.36	[−0.77, 0.05]	73%	
Personality disorders	4	0.20	[−0.15, 0.54]	21%		—				
Somatic symptom disorders	7	−0.63**	[−1.08, −0.17]	88%		7	−0.45**	[−0.77, −0.14]	73%	
Mixed/other	3	0.01	[−0.35, 0.36]	53%		—				
Treatment formats					0.61					0.13
Individual therapy	22	−0.12	[−0.34, 0.09]	69%		15	−0.43*	[−0.70, −0.16]	71%	
Group therapy	4	−0.37	[−0.96, 0.23]	87%		4	−0.32	[−0.87, 0.24]	85%	
Internet‐based treatment	—					—				
Age groups										
Adults	25	−0.18	[−0.39, 0.03]	75%		18	−0.39*	[−0.63, 0.15]	76%	
Children/adolescents	—					—				
Country					0.83					0.16
Western	20	−0.12	[−0.29, 0.05]	59%		15	−0.38**	[−0.65, −0.10]	74%	
Non‐western	7	−0.41	[−1.00, 0.18]	84%		4	−0.49	[−0.99, 0.01]	62%	

Abbreviations: CI, confidence interval; EDT, experiential dynamic therapy.

^a^ Direct comparisons between EDT and active medication protocols.

* *p* < 0.05.

** *p* < 0.01.

When analysing effects within specific diagnostic categories, the between‐subgroup test at post‐treatment was not significant (*p* = 0.07). However, while only on a trend level, effect sizes suggest that EDTs may outperform other active treatments in somatic symptom disorders (*g* = −0.63), though heterogeneity within the subgroup was high (*I* ^*2*^ = 88%). Egger's test was non‐significant, and there was no indication of outliers within subgroups. At follow‐up, the between‐subgroup test was significant, with effect sizes suggesting a significant and large effect for mood disorders (*g* = −1.01) and a significant small effect for somatic symptom disorders (*g* = −0.45). Heterogeneity was moderate, but the Egger's remained non‐significant across subgroups, and sensitivity analyses did not meaningfully change estimates. Post hoc *Q*‐tests indicated the effect for mood disorders was significantly larger than anxiety disorders (*Q* = 2.21, *p* = 0.027) and somatic symptom disorders (*Q* = 2.13, *p* = 0.033).

Comparisons between treatment formats included only individual therapy and group therapy, as only one study examined internet‐based EDT versus an active control. The between‐subgroup test was non‐significant at post‐treatment and follow‐up, indicating consistency in effects across examined formats. Although heterogeneity ranged from moderate to high, Egger's test was non‐significant, and no indication of publication bias or outlier influence was detected.

Subgroup analyses for age groups could not be performed, as only two studies examined EDTs against an active treatment in a child/adolescent samples, and only one study provided follow‐up data. The estimate for adults aligned with the overall effects and remained stable in sensitivity analyses.

For comparisons with active controls, there was no indication of significant effect size differences between Western and non‐Western studies at post‐treatment or follow‐up. The small effect favouring EDTs at follow‐up was observed in both subgroups but was only significant in Western studies, which remained robust in sensitivity analyses.

Meta‐regression analyses indicated that sample size was not significantly related to effect sizes at post‐treatment (*β* = 0.000; 95% CI [−0.002, 0.002]; *p* = 0.830; *k* = 27) or follow‐up (*β* = 0.002; 95% CI [0.000, 0.005]; *p* = 0.063; *k* = 19). Similarly, the number of provided EDT sessions was unrelated to effect sizes at post‐treatment (*β* = 0.017; 95% CI [−0.002, 0.038]; *p* = 0.094; *k* = 26) and follow‐up (*β* = 0.004; 95% CI [−0.023, 0.032]; *p* = 0.739; *k* = 19). The effects at follow‐up were also unrelated to the number of months passed after post‐treatment (*β* = 0.015; 95% CI [−0.011, 0.041]; *p* = 0.237; *k* = 19). However, as observed for inactive controls, year of publication was significantly associated with effects sizes at post‐treatment (*β* = −0.030; 95% CI [−0.046, −0.013]; *p* < 0.001; *k* = 27), but not at follow‐up (*β* = −0.018; 95% CI [−0.042, 0.006]; *p* = 0.130; *k* = 19).

### 3.7 Study Quality Analyses

The average study quality across all included studies was *M* = 25.6 (*SD* = 8.8; range = 11–44; Mdn = 27.0), surpassing the cut‐off of 24 for adequate quality on the RCT‐PQRS (Gerber et al. 2011). A significant difference was observed between Western (*k* = 38, *M* = 29.3, *SD* = 6.6) and non‐Western (*k* = 19, *M* = 18.3, *SD* = 7.9) studies, with Western studies generally rated as higher quality (*t* = −5.60, *df* = 56, *p* < 0.001).

Meta‐regression analyses revealed a significant association between higher study quality and smaller treatment effects compared to inactive controls at post‐treatment (*β* = 0.034, 95% CI [0.005, 0.063], *p* = 0.020, *k* = 41). A similar trend was observed at follow‐up (*β* = 0.039, 95% CI [−0.001, 0.079], *k* = 20), though it did not reach statistical significance (*p* = 0.058). In comparisons with active controls, study quality showed no significant relationship with effect sizes at post‐treatment (*β* = 0.005, 95% CI [−0.019, 0.029], *p* = 0.403, *k* = 27) or follow‐up (*β* = 0.017, 95% CI [−0.013, 0.048], *p* = 0.253, *k* = 19). Bubble plots for these analyses are available in the Supplement.

To further explore the impact of study quality, we conducted a post hoc subgroup analysis classifying studies as low quality (*k* = 23, 40.4%) if they had an RCT‐PQRS score of ≤ 23 and high quality (*k* = 34, 59.6%) if they scored ≥ 24. Compared to inactive controls, low‐quality studies showed larger absolute effect sizes than high‐quality studies at post‐treatment (*g* = −1.29 vs. *g* = −0.73; *Q*_*between*_ = 3.40, *p* = 0.065) and follow‐up (*g* = −1.48 vs. *g* = −0.79; *Q*_*between*_ = 3.16, *p* = 0.075), though these differences were not statistically significant. No significant differences between low‐ and high‐quality studies were found in comparisons to active controls at post‐treatment (*g* = −0.14 vs. *g* = −0.20; *Q*_*between*_ = 0.08, *p* = 0.784) or follow‐up (*g* = −0.41 vs. *g* = −0.40; *Q*_*between*_ = 0.00, *p* = 0.995).

## 4 Discussion

This review provides an updated evaluation of the efficacy of EDTs for common mental health conditions. Our searches yielded a total of 57 included trials, representing a twofold increase from our previous review (Lilliengren et al. 2016) and reflecting the growing research and clinical interest in this area over the past decade. Notably, there has been a substantial increase in EDT research conducted in Iran, with 16 new studies meeting our inclusion criteria and many more excluded due to the unavailability of full‐text publications in English. This rise may be influenced by the legacy of Habib Davanloo, a key figure in the development of EDTs, who had Iranian heritage and whose work may have contributed to the strong presence of EDT research in the region.

Our findings indicate that EDTs yield large effects compared to inactive controls at post‐treatment (*g* = −0.96) and follow‐up (*g* = −1.11), and comparable effects to other active treatments at post‐treatment (*g* = −0.17). Notably, a small but significant effect was observed against active controls at follow‐up (*g* = −0.40), suggesting a potential modest long‐term advantage of EDTs over other active treatments. The average dropout rate of 13% suggests that EDT is tolerated similarly to psychotherapy in general (Swift et al. 2017), and we found no indication of differences in dropout rates compared to active control conditions. These results remained robust in sensitivity analyses and provide general support for EDTs as evidence‐based transdiagnostic treatments.

However, substantial between‐study heterogeneity was observed, warranting cautious interpretation. Heterogeneity was expected given our broad inclusion criteria and the diversity in study methodologies, participant characteristics, treatment formats and cultural contexts. We applied conservative methods to account for this in our analyses; nevertheless, unexplained variability remains, limiting the interpretability of our findings. Notably, high within‐group variability in subgroup analyses reduced statistical power (Cuijpers et al. 2021), possibly obscuring moderator effects. The implications of our findings and potential sources of heterogeneity are discussed in more detail below.

### 4.1 EDTs in Comparison to Inactive Controls

The effects of EDTs against inactive controls were notably larger compared to our previous review (Lilliengren et al. 2016). This discrepancy may partly stem from the prior study's use of aggregated measures, which could have underestimated effects by diluting the impact of targeted symptom improvements within broader composite scores. The present review focused on primary outcome measures, which may be more sensitive to changes in the targeted conditions. Further, the addition of newer studies may have increased the estimates since we found a significant positive relationship between year of publication and reported effect sizes. This trend appears partly driven by the increase in non‐Western studies utilizing waitlist or no‐treatment control conditions. In line with this, study quality also emerged as a significant moderator of effect sizes at post‐treatment and most low‐quality studies were conducted in non‐Western contexts. Thus, methodological limitations—such as small sample sizes and reliance on waitlist controls—may have led to an overestimation of the overall effect against inactive controls.

Subgroup analyses also reflected this pattern, showing that EDTs had more moderate effects with less heterogeneity when compared to more rigorous inactive controls such as TAU or limited treatments. This aligns with meta‐analytic findings suggesting that psychotherapy effects tend to be inflated when compared to waitlist controls rather than TAU or active treatments (Cuijpers et al. 2024a). While our results indicate that EDTs are at least as effective as psychotherapy in general (Cuijpers et al. 2024b), the precise magnitude of their effects against inactive controls should therefore be interpreted with caution. Given ongoing debates about the validity of waitlist‐controlled designs in psychotherapy research (Cristea 2019; Cuijpers et al. 2018; Munder et al. 2019a, 2019b), future EDT studies should prioritize more stringent control conditions to ensure more consistent and reliable effect estimates.

Subgroup analyses indicated consistent effects across diagnostic categories, reinforcing the transdiagnostic applicability of EDTs. Effect sizes were large, except for personality disorders, where a more moderate effect was observed—mirroring findings from other psychotherapy reviews (e.g., Keefe et al. 2020). Notably, we found no new studies evaluating EDTs for personality disorders, despite some models being well‐suited for complex, treatment‐resistant cases (Abbass and Town 2025). However, several studies on other primary diagnoses included substantial proportions of patients with personality disorders (e.g., Town et al. 2017). This underscores EDTs' transdiagnostic potential in comorbid populations, with ongoing research likely to provide further insights (e.g., Daniëls et al. 2023). Encouragingly, research on EDTs for anxiety disorders has expanded, with our findings suggesting robust effects. However, half of the included studies (*k* = 7) focused on social anxiety disorder, highlighting the need for further research on other anxiety subtypes, such as panic disorder and generalized anxiety disorder (e.g., Lilliengren et al. 2017).

Unlike our previous review, which suggested larger pre‐post effects for individual therapy compared to group therapy, we did not find significant differences between treatment formats at any timepoint. This suggests that EDTs may be equally effective when delivered in individual, group or internet‐based formats. Notably, five new studies of internet‐based EDTs were identified, highlighting the growing research and clinical interest in digital mental health interventions (Andersson et al. 2019). However, given that all internet‐based EDT studies were conducted in Sweden by affiliated researchers, replications in other cultural contexts are needed to establish generalizability.

Lastly, we found no significant differences in treatment effects between child/adolescent and adult samples, although there were only three studies in the child/adolescent category. While this finding suggests that EDTs may be broadly applicable across age groups, more research is clearly needed in this area.

### 4.2 EDTs in Comparison to Other Treatments

Our findings revealed no significant differences between EDTs and other active treatments at post‐treatment, reinforcing the notion that EDTs are at least as effective as other evidence‐based therapies. This aligns with a recent umbrella review by Leichsenring et al. (2023), which did not find significant differences between psychodynamic therapies and other active treatments in general. Additionally, study quality was not associated with effect sizes against active controls, suggesting that the relative efficacy of EDTs compared to other treatments is robust across studies of varying methodological rigour.

Subgroup analyses were all non‐significant at post‐treatment, indicating consistent effects across comparison conditions, diagnostic groups, treatment formats and cultural contexts. Still, we observed a moderate and significant effect (*g* = −0.63) favouring EDTs over other treatments for somatic symptom disorders at post‐treatment. Though this finding should be interpreted cautiously given that the between‐group *Q*‐test was not statistically significant (*p* = 0.07), we believe it is noteworthy and aligns with the growing interest in emotion‐focused interventions for somatic distress (Frisch and Gündel 2023). This finding also aligns with theories suggesting that unresolved emotional conflicts may contribute to somatic symptom disorders and given the potential role of emotional processing in alleviating somatic distress (Maroti et al. 2022; Schnabel et al. 2022), EDTs may offer unique advantages for individuals struggling with such conditions. Future research should explore whether specific EDT mechanisms, such as defence restructuring and deepened emotional processing, drive these improvements.

The small but statistically significant follow‐up effect (*g* = −0.40) suggests a potential long‐term advantage of EDTs over other active treatments. Although the average follow‐up period was less than a year, the lack of an observed relationship between effect size and follow‐up duration suggests this effect could persist over time. These findings extend those of our previous review (Lilliengren et al. 2016), which only identified a long‐term advantage in comparisons with manualized supportive therapies. In contrast, the current study found no significant subgroup differences, suggesting the possibility of broader effects across treatment types, although the subgroup comparison with CBT yielded a non‐significant effect.

Notably, our findings differ from those of Kivlighan et al. (2015), who found no enduring benefit of psychodynamic therapies over other treatments in their longitudinal meta‐analysis. Several factors may help explain this divergence. First, our review focused specifically on EDTs, which may yield different outcomes than psychodynamic approaches more broadly. Second, our analysis includes more recent studies, which may differ in treatment implementation, study design, or sample characteristics compared to those included in earlier meta‐analyses. The follow‐up effect in our study appeared most pronounced for mood and somatic symptom disorders, though conclusions are limited by the small number of studies in these subgroups. Though encouraging, future research with longer follow‐up periods and larger samples is needed to clarify the durability of EDT effects compared to other treatments across diagnostic conditions.

While the exact mechanisms underlying this potential long‐term advantage remain unclear, emotion‐focused interventions may play a crucial role in sustaining psychotherapy benefits over time. Given that maladaptive emotion regulation is a core transdiagnostic process in mental health conditions (e.g., Aldao et al. 2016; Sheppes et al. 2015), it is possible that EDTs' focus on transforming emotional responses rather than suppressing or avoiding them facilitates long‐term improvements. Moreover, EDTs explicitly focus on deepening emotional experience and restructuring maladaptive defences within the context of a secure therapeutic attachment relationship (Abbass 2015; Frederickson 2013, 2020; Fosha 2000), may not only reduce symptoms but also alter enduring personality patterns. This broader restructuring process may contribute to more durable therapeutic gains compared to approaches that primarily target symptom management. This aligns with broader psychotherapy research suggesting that improved emotion regulation is not only a treatment target but also a key mechanism of change (Sønderland et al. 2023). While several emotional change processes have been linked to psychotherapy outcomes, their specific role in maintaining long‐term improvements following EDTs warrants further investigation.

Our overall findings also align with a recent meta‐analysis on transdiagnostic CBT (TD‐CBT; Schaeuffele et al. 2024), which demonstrated moderate to large effects compared to controls and sustained benefits over time. However, a direct comparison between EDTs and TD‐CBT remains unexplored, presenting an important direction for future research. Additionally, EDTs may inform the refinement of other therapeutic models. For example, Thoma and Abbass (2022) suggest that specific EDT techniques that focus on unprocessed emotions within the here‐and‐now therapeutic relationship could be integrated into process‐based CBT. Future studies should examine whether incorporating EDT techniques enhances emotion‐focused mechanisms in other treatments and improves clinical outcomes. More broadly, repeated assessments of theoretically derived process variables within both EDT and active or inactive comparators would be crucial for elucidating the therapeutic mechanisms underlying the short‐ and long‐term effects of EDTs (e.g., Zilcha‐Mano 2019).

### 4.3 Strengths and Limitations

This study has several strengths compared to our previous review. First, we were able to include twice as many studies, significantly expanding the evidence base and enhancing the robustness of our findings. Our broadened scope now incorporates somatic symptom disorders as well as child and adolescent populations, which were previously excluded. Additionally, by focusing on controlled effect sizes for primary outcomes, we ensured a more precise estimation of treatment effects. Notably, our results remained stable in sensitivity analyses, with minimal indications of publication bias or significant outlier influence. Despite the generally high levels of heterogeneity observed, all estimates were derived using random‐effects models, ensuring more conservative and generalizable results. Another strength is the systematic assessment of study quality using an established psychotherapy‐specific instrument, which demonstrated excellent reliability and calibration among raters. This methodological rigour enhances confidence in our overall findings and conclusions.

However, there are also several limitations that warrant mentioning. As noted, high levels of heterogeneity complicate definitive conclusions, particularly regarding the specific magnitude of effects for EDTs against inactive controls. Although approximately 60% of included studies met the RCT‐PQRS cut‐off for adequate quality, study quality varied considerably, with many studies exhibiting poor reporting practices, especially among non‐Western studies. It is important to acknowledge that lower quality ratings in these studies may, at least partly, reflect language‐related barriers and non‐adherence to Western reporting standards, rather than true methodological weaknesses. Nonetheless, improving study quality should remain a priority for future EDT research. Besides the use of more rigorous controls, one overlooked factor was therapist competence and treatment fidelity, which were inconsistently assessed and reported. Variability in therapist training, competence and adherence to EDT protocols may have contributed to the observed heterogeneity, but could not be assessed for this review.

Another important limitation with our review is the exclusion of 41 studies that lacked a full‐text English publication. The majority of these studies were conducted in Iran and involved comparisons of EDTs to waitlist controls, with English abstracts typically suggesting positive outcomes. Given our finding that EDT effects appear inflated when compared to waitlist controls in lower‐quality non‐Western studies, including these studies could have introduced additional bias. Thus, while this exclusion limits the comprehensiveness of our review, we believe it was justified to ensure a more consistent evaluation.

Furthermore, our ability to draw conclusions about certain populations and treatment formats remains constrained by the small number of studies in key subgroups, such as child and adolescent samples and internet‐based EDTs. These gaps highlight a critical need for further research in these areas. Similarly, while the average follow‐up period across studies was 8.9 months, the variation in follow‐up durations limits our ability to make strong claims about the long‐term efficacy of EDTs. Although no significant associations were found between follow‐up duration and effect sizes, this remains a notable limitation, warranting caution in interpreting long‐term effects.

Finally, some key study moderators were not fully accounted for, including researcher allegiance effects (Munder et al. 2013). Given that most study investigators were likely strong proponents of EDTs, this represents a potential source of bias that future research should address.

## 5 Conclusion

The findings of this review support EDTs as evidence‐based transdiagnostic treatments for common mental health conditions, with large effects compared to inactive controls, comparable effects to other active treatments at post‐treatment and some evidence suggesting potential long‐term advantages. The review also highlights the growing body of EDT research, including in non‐Western contexts, and its increasing application across diverse treatment formats and populations.

However, our results should be interpreted with caution due to significant heterogeneity, the exclusion of non‐English full‐text studies and variability in study methodologies and quality. Future research should prioritize stringent control conditions, clearer assessment of treatment fidelity, repeated evaluation of key process variables and long‐term follow‐ups to further clarify EDTs' effectiveness. More studies on child/adolescent samples are warranted as well as studies targeting anxiety disorders besides social anxiety. Given their effectiveness across diagnostic categories, EDTs may be particularly well‐suited for treating comorbid and complex cases, an area that also warrants further study. Additionally, investigating how EDT techniques can complement other therapeutic models, such as process‐based CBT, could help refine treatment approaches and improve outcomes for individuals struggling with emotional regulation difficulties. Advancing research in these areas will not only strengthen the empirical foundation of EDTs but also expand their potential applications in clinical practice, enhancing accessibility and impact for a wider range of patients.

## Conflicts of Interest

P. L. provides professional training and supervision in EDT. P. L. and D. M. receive royalties from book sales related to EDTs. All authors receive occasional honoraria for EDT lectures and presentations through engagements with both non‐profit and for‐profit professional organizations.

## Supporting information


**Data S1.** Supporting Information.

## Data Availability

Data sharing not applicable to this article as no datasets were generated or analyzed during the current study.
