# Supplementary material for: The Efficacy of Experiential Dynamic Therapies: A 10‐Year Systematic Review and Meta‐Analysis Update
Source: Clin Psychol Psychother. 2025 May 23;32(3):e70086. doi: 10.1002/cpp.70086 (PMC12102587; doi:10.1002/cpp.70086)
Supplement: Supplementary file 1 — Data S1. Supporting Information. [file CPP-32-e70086-s001.docx]

**SUPPLEMENT**

**The Efficacy of Experiential Dynamic Therapies: A 10-year Systematic Review and Meta-Analysis Update**

**Searches**

The latest searches for this review were conducted on December 30^th^ 2024.

The following search string was used in PubMed (https://pubmed.ncbi.nlm.nih.gov), Cochrane Library (https://www.cochranelibrary.com) and PsychINFO (https://www.proquest.com/psycinfo/):

(random* OR controlled OR trial OR study OR RCT) AND (psychodyn* OR psychoanaly* OR "dynamic psychotherapy" OR "dynamic therapy") AND (therapy OR psychotherapy OR intervention OR treatment) NOT (“case study” OR “case report” OR review)

For Google Scholar (https://scholar.google.com), we narrowed our search using this string:

("randomized" OR “randomised” OR “trial” OR “study”) AND (“psychodynamic” OR “psychoanalytic”) AND (“Malan” OR “Davanloo”) AND (“experiential” OR "short-term" OR "intensive" OR "ISTDP" OR "Accelerated" OR "AEDP" OR "Affect Phobia" OR “emotional awareness and expression therapy” OR “EAET”) AND (“psychotherapy” OR “treatment” OR “intervention”)

Results are presented in the flow-chart. All titles and abstracts have been screened for this review.

**Full references to included studies:**

Abbasi, M. N., & Manavipour, D. (2023). The Effectiveness of Intensive Short Term Dynamic Psychotherapy on Clinical Symptoms and Academic Performance in Adolescents with Oppositional Defiant Disorders. *Iranian Evolutionary and Educational Psychology Journal*, *5*(2), 134–144. doi: [10.61186/ieepj.5.2.134](https://doi.org/10.61186/ieepj.5.2.134)

Abbass, A., Sheldon, A., Gyra, J., & Kalpin, A. (2008). Intensive short-term dynamic psychotherapy for DSM-IV personality disorders: a randomized controlled trial. *The Journal of Nervous and Mental Disease, 196*(3), 211–6. <https://doi.org/0.1097/NMD.0b013e3181662ff0>

Ajilchi, B., Nejati, V., Town, J. M., Wilson, R., & Abbass, A. (2016). Effects of Intensive Short-Term Dynamic Psychotherapy on Depressive Symptoms and Executive Functioning in Major Depression. *The Journal of Nervous and Mental Disease*, *204*(7), 500–505. <https://doi.org/10.1097/NMD.0000000000000518>

Bressi, C., Porcellana, M., Marinaccio, P. M., Nocito, E. P., & Magri, L. (2010). Short-term psychodynamic psychotherapy versus treatment as usual for depressive and anxiety disorders: a randomized clinical trial of efficacy. *The Journal of Nervous and Mental Disease*, *198*(9), 647–652. <https://doi.org/10.1097/NMD.0b013e3181ef3ebb>

Brodaty, H., & Andrews, G. (1983). Brief psychotherapy in family practice. A controlled prospective intervention trial. *The British Journal of Psychiatry, 143*(1), 11–19. <https://doi.org/10.1192/bjp.143.1.11>

Bögels, S., Wijts, P., Oort, F., & Sallaerts, S. J. (2014). Psychodynamic psychotherapy versus cognitive behaviour therapy for social anxiety disorder: An efficacy and partial effectiveness trial. *Depression and Anxiety, 11*, 1–11. <https://doi.org/10.1002/da.22246>

Chavooshi, B., Mohammadkhani, P., & Dolatshahee, B. (2016a). Efficacy of Intensive Short-Term Dynamic Psychotherapy for Medically Unexplained Pain: A Pilot Three-Armed Randomized Controlled Trial Comparison with Mindfulness-Based Stress Reduction. *Psychotherapy and Psychosomatics*, *85*(2), 123–125. <https://doi.org/10.1159/000441698>

Chavooshi, B., Mohammadkhani, P., & Dolatshahi, B. (2016b). A Randomized Double-Blind Controlled Trial Comparing Davanloo Intensive Short-Term Dynamic Psychotherapy as Internet-Delivered Vs Treatment as Usual for Medically Unexplained Pain: A 6-Month Pilot Study. *Psychosomatics*, *57*(3), 292–300. <https://doi.org/10.1016/j.psym.2016.01.001>

Chavooshi, B., Saberi, M., Tavallaie, S. A., & Sahraei, H. (2017). Psychotherapy for Medically Unexplained Pain: A Randomized Clinical Trial Comparing Intensive Short-Term Dynamic Psychotherapy and Cognitive-Behavior Therapy. *Psychosomatics*, *58*(5), 506–518. <https://doi.org/10.1016/j.psym.2017.01.003>

Dare, C., Eisler, I., Russell, G., Treasure, J., & Dodge, L. (2001). Psychological therapies for adults with anorexia nervosa: randomised controlled trial of out-patient treatments. *The British Journal of Psychiatry : The Journal of Mental Science*, *178*, 216–221. <http://www.ncbi.nlm.nih.gov/pubmed/11230031>

Emmelkamp, P. M. G., Benner, A., Kuipers, A., Feiertag, G. A., Koster, H. C., & van Apeldoorn, F. J. (2006). Comparison of brief dynamic and cognitive-behavioural therapies in avoidant personality disorder. *The British Journal of Psychiatry: The Journal of Mental Science, 189*(1), 60–4. <https://doi.org/10.1192/bjp.bp.105.012153>

Farzadkia, M., Farhangi, A., & Abolghasemi, S. (2023). Comparison of Effectiveness of Intensive Short-Term Dynamic Psychotherapy and Mindfulness-Based Stress Reduction in Depression and Severity of Symptoms in Women with Fibromyalgia. *Jundishapur Journal of Chronic Disease Care*, *12*(3). <https://doi.org/10.5812/jjcdc-133803>

Hellerstein, D. J., Rosenthal, R. N., Pinsker, H., Samstag, L. W., Muran, J. C., & Winston, A. (1998). A randomized prospective study comparing supportive and dynamic therapies: Outcome and alliance. *Journal of Psychotherapy Practice & Research, 7*, 261–271.

Heshmati, R., Wienicke, F. J., & Driessen, E. (2023). The effects of intensive short-term dynamic psychotherapy on depressive symptoms, negative affect, and emotional repression in single treatment-resistant depression: A randomized controlled trial. *Psychotherapy (Chicago, Ill.)*. <https://doi.org/10.1037/pst0000500>

Johansson, R., Björklund, M., Hornborg, C., Karlsson, S., Hesser, H., Ljótsson, B., … Andersson, G. (2013). Affect-focused psychodynamic psychotherapy for depression and anxiety through the Internet: a randomized controlled trial. *PeerJ, 1:e102*. <https://doi.org/10.7717/peerj.102>

Johansson, R., Hesslow, T., Ljótsson, B., Jansson, A., Jonsson, L., Färdig, S., Karlsson, J., Hesser, H., Frederick, R. J., Lilliengren, P., Carlbring, P., & Andersson, G. (2017). Internet-based affect-focused psychodynamic therapy for social anxiety disorder: A randomized controlled trial with 2-year follow-up. *Psychotherapy*, *54*(4), 351–360. <https://doi.org/10.1037/pst0000147>

Knekt, P. & Lindfors, O. (Eds.) (2004). *A randomized trial of the effect of four forms of psychotherapy on depressive and anxiety disorders. Design, methods, and results on the effectiveness of short- term psychodynamic psychotherapy and solution-focused therapy during a one-year follow-up* [Sataunnaistettu kiininen koe neljan psykoterapiamuodon vaikuttavuudesta masennustiloihin ja ahdistuneisuushairioihin]. Helsinki: Studies in Social Security and Health, Vol. 77, issue 112.

Knekt, P., Lindfors, O., Harkanen, T., Valikoski, M., Virtala, E., Laaksonen, M. A., Marttunen, M., Kaipainen, M., & Renlund, C. (2008). Randomized trial on the effectiveness of long-and short-term psychodynamic psychotherapy and solution-focused therapy on psychiatric symptoms during a 3-year follow-up. *Psychological Medicine*, *38*(5), 689–703. <https://doi.org/10.1017/S003329170700164X>

Knijnik, D. Z., Kapczinski, F., Chachamovich, E., Margis, R., & Eizirik, C. L. (2004). Psychodynamic group treatment for generalized social phobia. *Revista Brasileira de Psiquiatria, 26*(2), 77–81. <https://doi.org/S1516-44462004000200003>

Knijnik, D. Z., Blanco, C., Salum, G. A., Moraes, C. U., Mombach, C., Almeida, E., … Eizirik, C. L. (2008). A pilot study of clonazepam versus psychodynamic group therapy plus clonazepam in the treatment of generalized social anxiety disorder. *European Psychiatry: The Journal of the Association of European Psychiatrists, 23*(8), 567–74. <https://doi.org/10.1016/j.eurpsy.2008.05.004>

Lindqvist, K., Mechler, J., Carlbring, P., Lilliengren, P., Falkenström, F., Andersson, G., Johansson, R., Edbrooke-Childs, J., Dahl, H. J., Lindert Bergsten, K., Midgley, N., Sandell, R., Thorén, A., Topooco, N., Ulberg, R., & Philips, B. (2020). Affect-Focused Psychodynamic Internet-Based Therapy for Adolescent Depression: Randomized Controlled Trial. *Journal of Medical Internet Research*, *22*(3), e18047. <https://doi.org/10.2196/18047>

Lumley, M. A., Schubiner, H., Lockhart, N. A., Kidwell, K. M., Harte, S. E., Clauw, D. J., & Williams, D. A. (2017). Emotional awareness and expression therapy, cognitive behavioral therapy, and education for fibromyalgia: A cluster-randomized controlled trial. *Pain*, *158*(12), 2354–2363. <https://doi.org/10.1097/j.pain.0000000000001036>

Maina, G., Forner, F., & Bogetto, F. (2005). Randomized controlled trial comparing brief dynamic and supportive therapy with waiting list condition in minor depressive disorders. *Psychotherapy and Psychosomatics, 74*(1), 43–50. <https://doi.org/10.1159/000082026>

Maina, G., Rosso, G., Crespi, C., & Bogetto, F. (2007). Combined brief dynamic therapy and pharmacotherapy in the treatment of major depressive disorder: a pilot study. *Psychotherapy and Psychosomatics, 76*(5), 298–305. <https://doi.oorg/10.1159/000104706>

Maina, G., Rosso, G., & Bogetto, F. (2009). Brief dynamic therapy combined with pharmacotherapy in the treatment of major depressive disorder: long-term results. *Journal of Affective Disorders, 114*(1-3), 200–7. <https://doi.org/10.1016/j.jad.2008.07.010>

Maina, G., Rosso, G., Rigardetto, S., Chiadò Piat, S., & Bogetto, F. (2010). No effect of adding brief dynamic therapy to pharmacotherapy in the treatment of obsessive-compulsive disorder with concurrent major depression. *Psychotherapy and Psychosomatics, 79*(5), 295–302. <https://doi.org10.1159/000318296>

Marmar, C. R., Horowitz, M. J., Weiss, D. S., Wilner, N. R., & Kaltreider, N. B. (1988). A controlled trial of brief psychotherapy and mutual-help group treatment of conjugal bereavement. *American Journal of Psychiatry, 145*(2), 203–209. <https://doi.org/10.1176/ajp.145.2.203>

Maroti, D., Lumley, M. A., Schubiner, H., Lilliengren, P., Bileviciute-Ljungar, I., Ljótsson, B., & Johansson, R. (2022). Internet-based emotional awareness and expression therapy for somatic symptom disorder: A randomized controlled trial. *Journal of Psychosomatic Research*, *163*(March), 111068. <https://doi.org/10.1016/j.jpsychores.2022.111068>

Martini, B., Rosso, G., Chiodelli, D. F., De Cori, D., & Maina, G. (2011). Brief dynamic therapy combined with pharmacotherapy in the treatment of panic disorder with concurrent depressive symptoms. *Clinical Neuropsychiatry, 8*(3), 204–211.

Mechler, J., Lindqvist, K., Carlbring, P., Topooco, N., Falkenström, F., Lilliengren, P., Andersson, G., Johansson, R., Midgley, N., Edbrooke-Childs, J., Dahl, H.-S. J., Sandell, R., Thorén, A., Ulberg, R., Bergsten, K. L., & Philips, B. (2022). Therapist-guided internet-based psychodynamic therapy versus cognitive behavioural therapy for adolescent depression in Sweden: a randomised, clinical, non-inferiority trial. *The Lancet Digital Health*, *4*(8), e594–e603. <https://doi.org/10.1016/S2589-7500(22)00095-4>

Mechler, J., Lindqvist, K., Magnusson, K., Ringström, A., Krafman, J. D., Alvinzi, P., Kassius, L., Sowa, J., Andersson, G., & Carlbring, P. (2024). Guided and unguided internet-delivered psychodynamic therapy for social anxiety disorder: A randomized controlled trial. *Npj Mental Health Research*, *3*(1), 1–10. <https://doi.org/10.1038/s44184-024-00063-0>

Milo, F., Imondi, C., D’Amore, C., Angelino, G., Knafelz, D., Bracci, F., Dall’Oglio, L., De Angelis, P., & Tabarini, P. (2023). Short-term Psychodynamic Psychotherapy in Addition to Standard Medical Therapy Increases Clinical Remission in Adolescents and Young Adults with Inflammatory Bowel Disease: a Randomised Controlled Trial. *Journal of Crohn’s and Colitis*, *August*, 1–8. <https://doi.org/10.1093/ecco-jcc/jjad145>

Mowlaie, M., Hajloo, N., Hashjin, G., Mikaeili, N., & Heidari, S. (2018). The Effectiveness of Empathic Dynamic Psychotherapy and Anxiety-Regulating Psychodynamic Therapy in Treating Adult Separation Anxiety Disorder. *Journal of Practice in Clinical Psychology*, *6*(3), 143–153. <https://ejop.psychopen.eu/article/view/1528>

Nader-Mohammadi Moghadam, M., Atef-Vahid, M.-K., Asgharnejad-Farid, A.-A., Shabani, A., & Lavasni, F. (2015). Short-term Dynamic Psychotherapy versus Sertraline in Treatment of Social Phobia. *Iranian Journal of Psychiatry and Behavioral Sciences*, *9*(2), 2–6. <https://doi.org/10.17795/ijpbs228>

Nakhaei Moghadam, R., Bahrainian, S. A., & Nasri, M. (2024). Effectiveness of Intensive Short-term Dynamic Psychotherapy on Attachment Styles, Somatization, and Health Anxiety in Patients with Chronic Pain. *Journal of Assessment and Research in Applied Counseling*, *6*(1), 142–149. <https://doi.org/10.61838/kman.jarac.6.1.16>

Orvati Aziz, M., Mehrinejad, S. A., Hashemian, K., & Paivastegar, M. (2020). Integrative therapy (short-term psychodynamic psychotherapy & cognitive-behavioral therapy) and cognitive-behavioral therapy in the treatment of generalized anxiety disorder: A randomized controlled trial. *Complementary Therapies in Clinical Practice*, *39*, 101122. <https://doi.org/10.1016/j.ctcp.2020.101122>

Pierloot, R., & Vinck, J. (1978). Differential outcome of short-term dynamic psychotherapy and systematic desensitization in the treatment of anxious out-patients: A preliminary report. *Psychologica Belgica, 18*(1), 87–98. <https://doi.org/10.5334/pb.626>

Piper, W. E., Azim, H. F., McCallum, M., & Joyce, A. S. (1990). Patient suitability and outcome in short-term individual psychotherapy. *Journal of Consulting and Clinical Psychology, 58*(4), 475–481. <https://doi.org/10.1037/0022-006X.58.4.475>

Prout, T. A., Rice, T., Chung, H., Gorokhovsky, Y., Murphy, S., & Hoffman, L. (2022). Randomized controlled trial of Regulation Focused Psychotherapy for children: A manualized psychodynamic treatment for externalizing behaviors. *Psychotherapy Research*, *32*(5), 555–570. <https://doi.org/10.1080/10503307.2021.1980626>

Rahmani, F., Rezaei, F., Nasuri, M., & Sc, M. (2011). Major Depressive Disorder: A Comparison of a Focused Psychodynamic Psychotherapy and Pharmacotherapy. *Iranian Journal of Psychiatry and Behavioral Sciences*, *5*(51), 37–44. <http://ijpbs.mazums.ac.ir/article-1-64-en.pdf>

Rahmani, F., Abbass, A., Hemmati, A., Ghaffari, N., & Rezaei Mirghaed, S. (2020a). Challenging the role of challenge in intensive short-term dynamic psychotherapy for social anxiety disorder: A randomized controlled trial. *Journal of Clinical Psychology*, 1–10. <https://doi.org/10.1002/jclp.22993>

Rahmani, F., Abbass, A., Hemmati, A., Mirghaed, S. R., & Ghaffari, N. (2020b). The Efficacy of Intensive Short-Term Dynamic Psychotherapy for Social Anxiety Disorder. *The Journal of Nervous and Mental Disease*, *208*(3), 245-251. <https://doi.org/10.1097/NMD.0000000000001097>

Reneses, B., Galián, M., Serrano, R., Figuera, D., Fernandez Del Moral, A., López-Ibor, J. J., … Trujillo, M. (2013). A new time limited psychotherapy for BPD: Preliminary results of a randomized and controlled trial. *Actas Españolas de Psiquiatría, 41*(3), 139–48.  PMID: 23803797

Rosso, G., Martini, B., & Maina, G. (2012). Brief dynamic therapy and depression severity: A single-blind, randomized study. *Journal of Affective Disorders*, 1–6. <https://doi.org/10.1016/j.jad.2012.10.017>

Rostami Ravari, A. M., Vaziri, S., Sarafraz, M., Branch, R., Branch, R., Capacity, P., Disorders, F. G., Sarafraz, M., Capacity, P., Severity, A., & Disorders, F. G. (2024). The Impact of Intensive Short-Term Dynamic Psychotherapy (ISTDP) on Psychological Capacity, Anxiety Severity, and Functional Gastrointestinal Disorders in Psychosomatic Patients with Gastrointestinal Symptoms. *International Journal of Education and Cognitive Sciences*, *5*(2), 101–111. <https://doi.org/https://doi.org/10.61838/kman.ijecs.5.2.12>

Salminen, J. K., Karlsson, H., Hietala, J., Kajander, J., Aalto, S., Markkula, J., … Toikka, T. (2008). Short-term psychodynamic psychotherapy and fluoxetine in major depressive disorder: a randomized comparative study. *Psychotherapy and Psychosomatics, 77*(6), 351–7. <https://doi.org/10.1159/000151388>

Shafiei, F., Dehghani, M., Lavasani, F. F., Manouchehri, M., & Mokhtare, M. (2024). Intensive short-term dynamic psychotherapy for irritable bowel syndrome: a randomized controlled trial examining improvements in emotion regulation, defense mechanisms, quality of life, and IBS symptoms. *Frontiers in Psychology*, *15*(March), 1–12. <https://doi.org/10.3389/fpsyg.2024.1293150>

Shahverdi, Z. A., Dehghani, M., Ashouri, A., Manouchehri, M., & Mohebi, N. (2024). Effectiveness of intensive short-term dynamic psychotherapy for Tension-Type Headache (TTH): A randomized controlled trial of effects on emotion regulation, anger, anxiety, and TTH symptom severity. *Acta Psychologica*, *244*(January), 104176. <https://doi.org/10.1016/j.actpsy.2024.104176>

Svartberg, M., Stiles, T. C., & Seltzer, M. H. (2004). Randomized, controlled trial of the effectiveness of short-term dynamic psychotherapy and cognitive therapy for cluster C personality disorders. *American Journal of Psychiatry, 161*(5), 810–817. <https://doi.org/10.1176/appi.ajp.161.5.810>

Tasca, G., Ritchie, K., Conrad, G., Balfour, L., Gayton, J., Lybanon, V., & Bissada, H. (2006). Attachment scales predict outcome in a randomized controlled trial of two group therapies for binge eating disorder: An aptitude by treatment interaction. *Psychotherapy Research, 16*(1), 106–121. <https://doi.org/10.1080/10503300500090928>

Thakur, E. R., Holmes, H. J., Lockhart, N. A., Carty, J. N., Ziadni, M. S., Doherty, H. K., Lackner, J. M., Schubiner, H., & Lumley, M. A. (2017). Emotional awareness and expression training improves irritable bowel syndrome: A randomized controlled trial. *Neurogastroenterology and Motility*, *29*(12), 1–10. <https://doi.org/10.1111/nmo.13143>

Town, J. M., Abbass, A., Stride, C., & Bernier, D. (2017). A randomised controlled trial of Intensive Short-Term Dynamic Psychotherapy for treatment resistant depression: the Halifax Depression Study. *Journal of Affective Disorders*, *214*(September 2016), 15–25. <https://doi.org/10.1016/j.jad.2017.02.035>

Town, J. M., Abbass, A., Stride, C., Nunes, A., Bernier, D., & Berrigan, P. (2020). Efficacy and cost-effectiveness of intensive short-term dynamic psychotherapy for treatment resistant depression: 18-Month follow-up of the Halifax depression trial. *Journal of Affective Disorders*, *273*(May), 194–202. <https://doi.org/10.1016/j.jad.2020.04.035>

Town, J. M., Abbass, A., & Campbell, S. (2024). Halifax somatic symptom disorder trial: A pilot randomized controlled trial of intensive short-term dynamic psychotherapy in the emergency department. *Journal of Psychosomatic Research*, *187*(April), 111889. <https://doi.org/10.1016/j.jpsychores.2024.111889>

Trowell, J., Joffe, I., Campbell, J., Clemente, C., Almqvist, F., Soininen, M., Koskenranta-Aalto, U., Weintraub, S., Kolaitis, G., Tomaras, V., Anastasopoulos, D., Grayson, K., Barnes, J., & Tsiantis, J. (2007). Childhood depression: a place for psychotherapy. An outcome study comparing individual psychodynamic psychotherapy and family therapy. *European Child & Adolescent Psychiatry*, *16*(3), 157–167. <https://doi.org/10.1007/s00787-006-0584-x>

Wiborg, I. M., & Dahl, A. A. (1996). Does brief dynamic psychotherapy reduce the relapse rate of panic disorder? *Archives of General Psychiatry, 53*(8), 689–694. <https://doi.org/10.1001/archpsyc.1996.01830080041008>

Winston, A., Laikin, M., Pollack, J., Samstag, L. W., McCullough, L., & Muran, J. C. (1994). Short-term psychotherapy of personality disorders. *The American Journal of Psychiatry, 151*(2), 190–194. <https://doi.org/10.1176/ajp.151.2.190>

Yarns, B. C., Lumley, M. A., Cassidy, J. T., Steers, W. N., Osato, S., Schubiner, H., & Sultzer, D. L. (2020). Emotional Awareness and Expression Therapy Achieves Greater Pain Reduction than Cognitive Behavioral Therapy in Older Adults with Chronic Musculoskeletal Pain: A Preliminary Randomized Comparison Trial. *Pain Medicine*, *0*(0), 1–13. <https://doi.org/10.1093/pm/pnaa145>

Yarns, B. C., Jackson, N. J., Alas, A., Melrose, R. J., Lumley, M. A., & Sultzer, D. L. (2024). Emotional Awareness and Expression Therapy vs Cognitive Behavioral Therapy for Chronic Pain in Older Veterans. *JAMA Network Open*, *7*(6), e2415842. <https://doi.org/10.1001/jamanetworkopen.2024.15842>

Ziapour, A., Hajiazizi, A., Ahmadi, M., & Dehghan, F. (2023). Effect of short‐term dynamic psychotherapy on sexual function and marital satisfaction in women with depression: Clinical trial study. *Health Science Reports*, *6*(6). <https://doi.org/10.1002/hsr2.1370>

**Excluded studies:**

| Reference | Reason for exclusion |
| --- | --- |
| Afrooz, G., Hashemian, K., & Bagheri, M. (2023). Comparing Effectiveness of Short Term Psychodynamic Psychotherapy and Emotion-Focused Couple Therapy on Couples with Alexithymia. *Rooyesh-e-Ravanshenasi Journal (RRJ)*, *12*(4), 1-14. <https://frooyesh.ir/browse.php?a_id=3877&slc_lang=en&sid=1&printcase=1&hbnr=1&hmb=1> | No full-text in English. |
| Ahmadi, F., Goodarzi, M., & Kazemi rezaei, S. A. (2021). the Effectiveness of Intensive Short-Term Dynamic Psychotherapy in dissociative experiences, object relations and delayed reward discounting in patients with substance use disorders and addiction. Journal of Clinical Psychology, 13(1), 65-78. doi: [10.22075/jcp.2021.21041.1931](https://jcp.semnan.ac.ir/article_5059_en.html) | No full-text in English. |
| Ahmadi, F., Amjad, F. V., Rezaei, S. A. K., & Mohammadi, S. (2021). The Effectiveness of Intensive Short-Term Dynamic Psychotherapy ISTDP in symptoms depression, anxiety, posttraumatic stress and guilt felling in Bereaved people from the Disease COVID-19. *Quarterly Journal of Nursing Management*, *10*(3). <https://www.sid.ir/paper/1010719/en> | No full-text in English. |
| Alinasab, S., Hamid, N., & Mirhashemy, M. (2025). Comparing the effectiveness of Emotional Awareness and Expression Therapy with Acceptance and Commitment Therapy on Anger and Pain Intensity among Women with Breast Cancer. *Iranian Journal of Breast Diseases*, *17*(4), 117–135. [10.61186/ijbd.17.4.117](http://dx.doi.org/10.61186/ijbd.17.4.117) | No full-text in English. |
| Alirezaee, S., Nia, K. S., & Akbari, H. (2023). Comparison of the effectiveness of cognitive-behavioral psychotherapy and intensive short-term dynamic psychotherapy on self-compassion in cancer patients. *EBNESINA, 24*(4), 43-53. <http://ebnesina.ajaums.ac.ir/article-1-1157-fa.pdf> | No full-text in English. |
| Alirezaee, S., Shariatnia, K., & Akbari, H. (2023). Comparing the Effectiveness Cognitive-behavioral Therapy and Intensive Short-term Dynamic Psychotherapy in Reducing Death Anxiety. *Journal of Preventive Medicine*, *9*(4), 390–401. <https://jpm.hums.ac.ir/article-1-675-en.pdf> | No full-text in English. |
| Amani, N., Haji alizadeh, K., Zarei, E., & Dortaj, F. (2020). Effectiveness of Intensive Short- Term Dynamic Psychotherapy on Anxiety in Rheumatoid Arthritis Patients. *Journal of Research in Behavioural Sciences*, *18*(3), 349–358. <https://doi.org/10.52547/rbs.18.3.349> | No full-text in English. |
| Aminifar, S., Bahrami Hidaji, M., Kraskian Mujembar, A., Mansoobifar, M., Peyvandi, P., & 1. (2023). The effectiveness of short-term intensive psychodynamic therapy on emotional self-awareness, empathy and self-compassion in psychotherapy trainees. *Adolescent And Youth Psychological Studies*, *4*(5), 103–115. <https://journals.kmanpub.com/index.php/jayps/article/download/1166/1381> | Non-clinical sample. |
| Arabkhazaeli, F., & Ghorbanzadeh, M. (2024). The Effectiveness of Intensive Short-Term Dynamic Psychotherapy on Pain Perception and Pain Catastrophizing in Women with Breast Cancer. *Applied Family Therapy Journal*, *5*(4), 133–139. <https://doi.org/10.61838/kman.aftj.5.4.15> | Not clear if EDT and not clear if individual or group therapy was provided. Author could not be reached. |
| Bahremand M, Talebzadeh Shoushtari M, Marashian F S. Effectiveness of Intensive Short-Term Dynamic Psychotherapy and Existential Therapy on Self-Compassion and Existential Anxiety in Infertile Individuals. *Avicenna J Neuro Psycho Physiology 2024; 11* (2) :50-56 URL: <http://ajnpp.umsha.ac.ir/article-1-489-en.html> | Non-clinical sample. |
| Baldoni, F., Baldaro, B., & Trombini, G. (1995). Psychotherapeutic perspectives in urethral syndrome. *Stress Medicine*, *11*(July 1994), 79–84.  <https://doi.org/10.1002/smi.2460110115> | No data to calculate effect sizes. |
| Ben-Itzhak, S., Bluvstein, I., Schreiber, S., Aharonov-Zaig, I., Maor, M., Lipnik, R., & Bloch, M. (2012). The effectiveness of brief versus intermediate duration psychodynamic psychotherapy in the treatment of adjustment disorder. *Journal of Contemporary Psychotherapy, 42*(4), 249–256. <https://doi.org/10.1007/s10879-012-9208-6> | Parametric RCT. |
| Bloch, M., Meiboom, H., Lorberblatt, M., Bluvstein, I., Aharonov, I., & Schreiber, S. (2012). The effect of sertraline add-on to brief dynamic psychotherapy for the treatment of postpartum depression: a randomized, double-blind, placebo-controlled study. *The Journal of Clinical Psychiatry, 73*(2), 235–41. <https://doi.org/10.4088/JCP.11m07117> | Parametric RCT. |
| Carty, J. N., Ziadni, M. S., Holmes, H. J., Tomakowsky, J., Peters, K., Schubiner, H., & Lumley, M. A. (2019). The Effects of a Life Stress Emotional Awareness and Expression Interview for Women with Chronic Urogenital Pain: A Randomized Controlled Trial. *Pain Medicine*, *20*(7), 1321–1329. <https://doi.org/10.1093/pm/pny182> | Single-session intervention. |
| Chavooshi, B., Mohammadkhani, P., & Dolatshahee, B. (2016). A Randomized Double- Blind Controlled Trial Comparing Davanloo’s Intensive Short-Term Dynamic Psychotherapy as Internet-Delivered Versus Treatment as Usual for Medically Unexplained Pain: A Six- Month Pilot Study. *Psychosomatics, 57*(3), 292–300. [http://linkinghub.elsevier.com/retrieve/pii/S0 033318216000025](http://linkinghub.elsevier.com/retrieve/pii/S0%20033318216000025) | Parametric RCT. |
| Ezzatpour, M., Tanah, Z., Amraei, K., & Goodarzi, K. (2024). Comparing the Effectiveness of Acceptance and Commitment-based Therapy and Intensive Short-term Dynamic Psychotherapy on Ego strength in women with Obsessive Compulsive Disorder. *Family and Health*, *14*(1), 69–87. <https://journal.astara.ir/article_712950.html> | Not clear if random allocation was used. Author could not be reached. |
| Ezzatpour M, Tanah Z, Amraei K, Goodarzi K. Comparison of the Effectiveness of Acceptance and Commitment-Based Therapy and Intensive Short-term Dynamic Psychotherapy on Perfectionism and Quality of Life in Patients with Obsessive Compulsive Disorder. *Avicenna J Neuro Psycho Physiology 2023; 10* (4) :164-173 URL: <http://ajnpp.umsha.ac.ir/article-1-478-en.html> | Not clear if random allocation was used. Author could not be reached. |
| Farahdel, S., Mahdian, H., & Ghasemi Motlagh, M. (2023). Comparing the Effectiveness of Intensive Short-Term Dynamic Psychotherapy (ISTDP) and Acceptance and Commitment Therapy Enriched with Compassion on the Components of Marital burnout in Women Seeking Divorce. *The Psychology of Woman Journal*, *4*(2), 62–72. <https://doi.org/10.61838/kman.pwj.4.2.8> | Non-clinical sample. |
| Farshi Sotoudeh, A., Esmkhani Akbarinejhad, H., & Faroughi, P. (2024). Effectiveness of Intensive Short-Term Dynamic Psychotherapy on the Meaning of Life and Death Anxiety of Bereaved Mothers. *Community Health Journal 2024*, *18*(2), 73–84. <https://chj.rums.ac.ir/article_211465.html> | No full-text in English. |
| Farzdi, H., Heidarei, A., Moradimanesh, F., & Naderi, F. (2021). The Effectiveness of Short-Term Dynamic Psychotherapy on Symptoms Severity and Disease Perception among Patients with Irritable Bowel Syndrome : A Pilot study. *Salāmat-i Ijtim (Community Health)*, *8*(2), 221–231. <https://journals.sbmu.ac.ir/ch/index.php/ch/article/download/31425/20424/125221> | No data to calculate effect sizes. |
| Fooladi, F., Broojeni, M. K., Soodjani, Y. R., & Province, B. (2018). Effectiveness of Intensive Short-Term Dynamic Psychotherapy (ISTDP) on the Social Anxiety of Mothers of Children with Asperger Syndrome. *Journal of Exceptional Children (Iran)*, 55–64. <https://joec.ir/browse.php?a_id=645&sid=1&slc_lang=en&ftxt=1> | No full-text in English. |
| Frederickson, J., Dendooven, B., Abbass, A., Solbakken, O. A., & Rousmaniere, T. (2019). Pilot study: An inpatient drug rehabilitation program based on intensive short-term dynamic psychotherapy. *Journal of Addictive Diseases*, *0*(0), 1–7. <https://doi.org/10.1080/10550887.2019.1658513> | Inpatient treatment program. |
| Ghorbani, N., Dadsetan, P., Ejei, J., & Motiyan, H. (2000). The consequences of overcoming resistance and emotional disclosure on lymphocyte T-helper and T-supressor and psychological pathology. *Journal of Psychology (Persian), 3*, 368– 389. | No full-text in English. |
| Habiba, M., & Arab, A. (2023). The effectiveness Intensive Short-Term Dynamic Psychotherapy in Defensive style changes and Emotional cognitive cohesion In patients with depression. *Iranian Journal of Neurodevelopmental Disorders*, *2*(1), 26-35. <https://maherpub.com/jndd/article/view/22> | No full-text in English. |
| Hakami, M. S., Mosayebi, M., Jafari, A., & Arefi, F. (2024). The effectiveness of Intensive Short-term Dynamic Psychotherapy (ISTDP) on Emotional Suppression and Severity of Intestinal Symptoms of IBS-D Patients with Comorbid Depression. *Medical Journal of Mashhad University of Medical Sciences*, *67*(4). <https://doi.org/10.22038/MJMS.2024.25024> | No full-text in English. |
| Harchegani, N. B., & Ghazanfari, A. (2024). Comparison of The Effectiveness of Intensive Short-term Dynamic Psychotherapy (ISTDP) and Schema Therapy on Mothers of 5-6-year-old Children with Separation Anxiety. Razavi International Journal of Medicin, 12(2), e1298. <https://doi.org/10.30483/RIJM.2024.254491.1298> | Non-clinical sample. |
| Harnashki, H. K., Ahadi, H., & Tajeri, D. (2021). The Effectiveness of Short-term Intensive Dynamic Psychotherapy Interventions and Twelve-step Method in Reducing Drug Temptation and Reducing the Projective Defense Mechanisms of Recovering Addicts in Tehran Province. *Journal of Preventive Counselling*, *2*(4), 18–31. <https://jpc.uma.ac.ir/article_1612_d2e5ca82630ebc190f8c85e4cfa95503.pdf> | No measure for primary outcome. |
| Hatami H, Mohammadi N, Hadian Fard H, Aflakseir A A. the effectiveness of schema therapy (ST) and intensive short-term dynamic psychotherapy (ISTDP) for improving emotion regulation in Complex PTSD (CPTSD). *Journal title 2024; 18* (3) : 4 <http://rph.khu.ac.ir/article-1-4472-en.html> | No full-text in English. |
| Heidarinasab L, Khoryanian M, Tabibi Z. Effectiveness of intensive short‐term dynamic psychotherapy in reducing symptoms and changing defense styles in patients with depression. *J Clin Psychol Stud.* 2014;14:143‐167. <https://jcps.atu.ac.ir/article_517_en.html> | No full-text in English. |
| Irani, Z., Khakppour, R., & Behboodi, M. (2024). Comparison of the effectiveness of intensive short-term dynamic psychotherapy ( ISTDP ) and existential therapy on the signs and symptoms of psychosomatic patients referred to Imam Khomeini Hospital in Tehran. *Medical Science Journal of Islamic Azad University, 33*(4), 393–404.  <http://tmuj.iautmu.ac.ir/article-1-2099-en.html> | No full-text in English. |
| Jafarian Nemini, F., Shojaedin, A., Ghorbani, N., & Rostami, R. (2020). Efficacy of acupuncture laser with short-term intensive dynamic psychotherapy in the treatment of depression: a pilot study. *Journal of psychologicalscience*, *19*(87), 265-273. <http://psychologicalscience.ir/browse.php?a_id=655&slc_lang=en&sid=1&printcase=1&hbnr=1&hmb=1> | No full-text in English. |
| Jafari Soheil, Joharifard Reza. The Effectiveness of Intensive Short-Term Dynamic Psychotherapy on the Alexithymia, Defense Styles, and Ego Strength in Patients with Irritable Bowel Syndrome: A Quasi-Experimental Study. J Rafsanjan Univ Med Sci 2023; 22 (3): 243-58.  <http://journal.rums.ac.ir/article-1-6877-en.html> | No full-text in English. |
| Jamali S, Mehrabizeh Honarmand M, Hashemi S E, Davoodi I. The Effectiveness of "Intensive and Short-Term Psychodynamic Therapy" on Fear of Guilt and Latent Aggression in Female Patients with Contamination Obsessive-Compulsive Disorder. JHPM 2022; 11 (1) :47-60 URL: <http://jhpm.ir/article-1-1256-en.html> | No full-text in English. |
| Kafee Hernashki, H. , Ahadi, H. and Tajeri, B. (2021). The effectiveness of short-term intensive Dynamic psychotherapy Interventions and twelve-step method in reducing drug temptation and reducing the Projective defense mechanisms of recovering addicts in Tehran province. Preventive Counseling, 2(4), 47-60. <https://jpc.uma.ac.ir/article_1612.html> | No data on primary outcome. |
| Karimi, M., Beliad, M. R., Peymani, J., Havassi Somar, N., & Jian Bagheri, M. (2023). Comparing the Effectiveness of Intensive Short-Term Dynamic Psychotherapy with Mindfulness-Based Cognitive Therapy on Anxiety Sensitivity and Its Dimensions in Women with Chronic Pain. *Community Health Journal (Persian)*, *17*(3), 1–13. <https://chj.rums.ac.ir/article_187141_en.html> | No full-text in English. |
| Kashefi, F., Azkhosh, M., & Khanjani, M. S. (2023). The Effectiveness of Intensive Short-Term Dynamic Psychotherapy on Self-Differentiation and Attachment Behavior in Couple Relationships of Women with Tendency to Marital Infidelity. *Iranian Journal of Psychiatric Nursing (Persian)*, *10*(6). <http://ijpn.ir/browse.php?a_code=A-10-2109-1&sid=1&slc_lang=en> | No full-text in English. |
| Kashfi, N., Ghanifar, H. M., Nasri, M., & Dastjerdi, G. (2022). The Effectiveness of Short-Term Intensive Dynamic Psychotherapy on Emotion Regulation and Prevention of Relapse of Recovering Addicts. *Journal off Modern Psychological Research (Iranian)*, *17*(68). <https://doi.org/10.22034/JMPR.2023.15330> | No full-text in English. |
| Khatami, M., & Sedaghati Fard, M. (2022). The effect of short-term intensive dynamic psychotherapy (ISTDP) on reducing anxiety, increasing self-empathy, coping and social adaptation in women with the experience of sexual assault. *Applied Family Therapy Journal (AFTJ)*, *3*(5), 661-677. <https://doi.org/10.61838/kman.aftj.3.5.39> | No full-text in English. |
| Kompoliti, K., Wilson, B., Stebbins, G., Bernard, B., & Hinson, V. (2013). Immediate vs. delayed treatment of psychogenic movement disorders with short term psychodynamic psychotherapy: Randomized clinical trial. *Parkinsonism & Related Disorders*, 7–10. <http://doi.org/10.1016/j.parkreldis.2013.09.018> | No data to calculate effect sizes. |
| Krohner, S., Town, J., Cannoy, C. N., Schubiner, H., Rapport, L. J., Grekin, E., & Lumley, M. A. (2023). Emotion-focused Psychodynamic Interview for People with Chronic Musculoskeletal Pain and Childhood Adversity: A Randomized Controlled Trial. *The Journal of Pain*, 100924. <https://doi.org/10.1016/j.jpain.2023.07.017> | Single-session intervention. |
| Lerner, A., Sigal, M., Bacalu, A., & Gelkopf, M. (1992). Short term versus long term psychotherapy in opioid dependence: A pilot study. *Israeli Journal of Psychiatry and Related Sciences, 29*(2), 114–9. <http://www.ncbi.nlm.nih.gov/pubmed/1526756> | Parametric RCT. |
| Linnet, J., & Jemec, G. B. (2001). Anxiety level and severity of skin condition predicts outcome of psychotherapy in atopic dermatitis patients. *International Journal of Dermatology*, *40*(10), 632–6. <https://doi.org/10.1046/j.1365-4362.2001.01272.x> | No data to calculate effect sizes. |
| Ludwig, G., Krenz, S., Zdrojewski, C., Bot, M., Rousselle, I., Stagno, D., … Stiefel, F. (2013). Psychodynamic interventions in cancer care I: psychometric results of a randomized controlled trial. *Psycho-Oncology*, (July 2009). <https://doi.org/10.1002/pon.3374> | Unclear if clinical sample in terms of psychiatric symptoms. |
| Mehboodi, K., Mohammadi, N., Rahimi, C., & Sarafraz, M. R. (2022). The efficacy of intensive short-term dynamic psychotherapy (ISTDP) on self-esteem, emotion regulation, and defense mechanisms in men with social anxiety disorder. *The Journal Of Psychological Science*, *21*(111), 461–474. <https://doi.org/10.52547/JPS.21.111.461> | No full-text in English. |
| Mirzai, F., Nikbakht, N., Norouziani, Z., Abdollahi-Chirani, E., Fathy-Karkaragh, F., & Mazroei, P. (2024). Reframing Self-Perception: Efficacy of Intensive Short-Term Dynamic Psychotherapy (ISTDP) on Self-Disgust, Physical Appearance Perfectionism, and Self-Esteem in Girls with Anorexia Nervosa. Int J Body Mind Cult. 11(5):514–524. *International Journal of Body, Mind and Culture*, *11*(5), 515–524. | Not clear if random allocation was used. |
| Mirzaei, A., & Sharifi, T. (2024). Comparison of the Effectiveness of Emotion-Focused Therapy and Intensive Short-Term Psychodynamic Therapy on Sexual Dysfunction and Quality of Sexual Life in Women. *Applied Family Therapy Journal*, *5*(2), 171–181. <https://doi.org/10.61838/kman.aftj.5.2.19> | Non-clinical sample. |
| Moharer, G. S., & Harafteh, F. S. K. (2021). The effect of intensive short-term dynamic psychotherapy on emotion regulation and health hardiness in patients with type 2 diabetes. *Journal of Consulting Excellence and Psychotherapy: Issue*, *10*, 1-111. <https://journals.iau.ir/article_683745_a0f1a3298a89edf1848e084e10aa1074.pdf> | No full-text in English. |
| Moradzadeh Khorasani, L., Mirzaian, B., & Hassanzadeh, R. (2020). The effectiveness of intensive short-term dynamic psychotherapy on mentalization in non-suicidal self-injurious people. *Iranian Journal of Psychiatric Nursing*, *8*(3), 22-35. <http://ijpn.ir/article-1-1561-en.pdf> | No full-text in English. |
| Mousavi Z, Naji A A. (2022). Effectiveness of Intensive and Short-Term Dynamic Psychotherapy (ISTDP) on Defense Mechanisms and Emotional Expression in individuals with Coronavirus-induced Death Anxiety.  Rooyesh. 11(2), 13-22.  URL: <http://frooyesh.ir/article-1-3315-en.html> | No full-text in English. |
| Nabizadeh, A., Farhadi, M., Rashid, K., & Kordnoghabi, R. (2019). The Effectiveness of Tactical Defensive Neutralization in Intensive Short-Term Dynamic Psychotherapy on Defensive Styles, Anxiety, and Fear of Intimacy in Non-Clinical Sample. *Journal of Research in Psychological Health (Persian)*, *13*(1), 24–39. <https://www.sid.ir/FileServer/JF/45113980102.pdf> | Non-clinical sample. |
| Nasseri, A., Khosrojavid, M., & Kafi Masouleh, S. M. (2024). Effect of Intensive Dovanlo’s short-term Daynamic Psychotrapy onThe Quality of Object Relations and Defense Mechanisms and in Adolescents White Separation Anxiety Disorder. Psychology of Exceptional Individuals, 14(53), 149–178. <https://doi.org/10.22054/JPE.2023.70870.2510> | No full-text in English. |
| Pakdel, H., Shohrabi, F., & Haji Alizadeh, K. (2022). The efficacy of intensive short-term dynamic psychotherapy on job stress coping strategies, health-related quality of life, and self-efficacy of iran air traffic controler staff. *Monthly Journal of Psychological Science*, *21*(111), 543–558. <https://psychologicalscience.ir/article-1-1554-fa.html> | No full-text in English. |
| Parisuz, A., Najarpourian, S., & Mohammadi, K. (2019). The Effect of Intensive Short-Term Dynamic Psychotherapy on Marital Conflicts and Interpersonal Processing of Married Women with Emotional Divorce. *Iranian Evolutionary and Educational Psychology Journal*, *1*(2), 112–122. <https://doi.org/10.29252/ieepj.1.2.112> | Non-clinical sample. |
| Pasbani Ardabili, M. , Borjali, A. and Pezeshk, S. (2018). The Effectiveness of Intensive Short-Term Dynamic Psychotherapy to Improve the Conflictive the Relationship between Mother-Child. Clinical Psychology Studies, 9(30), 139-162. doi: 10.22054/jcps.2018.8612 | No full-text in English. |
| Piper, W. E., Debbane, E. G., Bienvenu, J. P., & Garant, J. (1984). A comparative study of four forms of psychotherapy. *Journal of Consulting and Clinical Psychology*, *52*(2), 268–79. [https://doi.org/10.1037/0022-006X.52.2.268](https://psycnet.apa.org/doi/10.1037/0022-006X.52.2.268) | No data to calculate effect sizes. |
| Qaziani, M. M., & Arefi, M. (2017). The effectiveness of intensive short-term dynamic psychotherapy on affection control of anxious women. *International Journal of Educational and Psychological Researches*, *3*(4). <https://doi.org/10.4103/jepr.jepr> | Non-clinical sample. |
| Ranjbar Bahadori S, Taklavi S, Kazemi R. (2022). The effectiveness of intensive short-term dynamic psychotherapy on emotional expressiveness and differentiation betrayed women.  Rooyesh. 11(1), 113-124.  URL: <http://frooyesh.ir/article-1-3219-en.html> | Non-clinical sample. |
| Ranjbar Sudejani, Y., Sharifi, K., Sayyah, S. S., & Malek Mohamadi Galeh, Z. (2017). Effectiveness of Intensive Short-Term Dynamic Psychotherapy (ISTDP) on Correcting Irrational Beliefs and Reducing Cognitive Avoidance of People with Obsessive Compulsive Disorder. *Journal of Counseling Research [Iran]*, *62*(15), 140–169. <http://irancounseling.ir/journal/article-1-364-en.html> | No full-text in English. |
| Ranjbar Sudejani, Y., & Sharifi, K. (2017). Effectiveness of Intensive Short Term Dynamic Psychotherapy (ISTDP) on Social Anxiety of Children with Mental Disability and Visual Perception Disorder. *Exceptional Education*, *1*(144), 15–22. <http://exceptionaleducation.ir/article-1-947-en.html> | No full-text in English. |
| Rosser, R., Denford, J., Heslop, a, Kinston, W., Macklin, D., Minty, K., … Guz, a. (1983). Breathlessness and psychiatric morbidity in chronic bronchitis and emphysema: a study of psychotherapeutic management. *Psychological Medicine*, *13*(1), 93–110. <https://doi.org/10.1017/S0033291700050108> | No data to calculate effect sizes. |
| Salehian, N. (2022). The effect of intensive short-term dynamic psychotherapy (ISTDP) on reducing psychological symptoms in patients with histrionic personality (HPD). *JNPI*, *13*(17), 1–30. <http://jnip.ir/article-1-806-en.html> | No full-text in English. |
| Sarlaki, A., Farokhzad, M., Khanzadeh, F., Younesi sinaki, M., Razavi Mahdiian, S. Z., & Tayyar Parvin, M. (2024). Effectiveness of Intensive Short-Term Dynamic Psychotherapy on Object Relations, Anger, and Guilt in Women with Major Depressive Disorder. Psychological Achievements, (), -. <https://doi.org/10.22055/psy.2024.44694.3112> | No full-text in English. |
| Shams, S., Ghelichkhan, N., Yousefi, N., & Alijani, S. (2022). Comparison of the Effectiveness of Short-term Intensive Dynamic Psychotherapy and Metallization-based Therapy on Emotional Dysregulation , Defense Mechanisms and Insecure Attachment Styles in Women Who Have Experienced the Trauma of Marital Infidelity. *Iranian Journal of Psychiatric Nursing*, *10*(1). <https://ijpn.ir/files/site1/user_files_38e7a3/eng/sarashams-A-10-1782-5-ea500a4.pdf> | No full-text in English. |
| Sjödin, I., Svedlund, J., Ottoson, J. o., & Dotevall, G. (1986). Controlled study of psychotherapy in chronic peptic ulcer disease. *Psychosomatics*, *27*(3), 187–197. <https://doi.org/10.1016/S0033-3182(86)72711-4> | No data to calculate effect sizes. |
| Soufi Amlashi R, Shomali Oskoei A, Mirzaei K. The effectiveness of intensive short-term dynamic psychotherapy on emotion regulation difficulty in depressed people. *Shenakht Journal of Psychology and Psychiatry* 2024; 11 (4) :32-48 <http://shenakht.muk.ac.ir/article-1-2017-en.html> | No full-text in English. |
| Soufi Amlashi, R., Shomali Oskoei, A., & Mirzaei, K. (2023). The Effectiveness of Intensive Short-Term Dynamic Psychotherapy (ISTDP) on Self-Compassion and Defense Mechanisms in Individuals with Depression. *Journal of Assessment and Research in Applied Counseling*, *5*(3), 156–166. <https://doi.org/10.61838/kman.jarac.5.3.20> | No measure of primary outcome. Not clear which assessment point is used in analyses. |
| Svedlund, J., Ottosson, J.-O., Sjödin, I., & Dotewall, G. (1983). Controlled Study of Psychotherapy in Irritable Bowel Syndrome. *Lancet, 10*, 589–592. <https://doi.org/10.1053/j.gastro.2011.12.028> | No data to calculate effect sizes. |
| Taghavi, F., Mirzaie, P., Azandariani, M., & Rezaei Shojaei, S. (2020). The Effectiveness of Short-Term Dynamic Psychotherapy on Anxiety, Depression and Happiness in Women with Generalized Anxiety. *Medical Journal of Mashhad University of Medical Sciences*, *82*, 456–466. <https://mjms.mums.ac.ir/article_15075_en.html> | No full-text in English. |
| Tourani, L., Sharifi Daramadi, P., & Farrokhi, N. (2020). Effects of Intensive Short-Term Dynamic Psychotherapy on Depression and Anxiety in Derelict Adolescents. *MEJDS (Persian)*, *Nov*(10:173). | No full-text in English. |
| Yousefi, T., Mehabii, S., Hajrezaei, M. R., Kalantari, S., & Abolpour, E. (2022). Effectiveness of Dynamic Psychotherapy on Personality Organization and Self- Compassion in Patients with Psychosomatic Disorders. *Journal of Research in Behavioral Sciences*, *22*(1), 104–113. <http://rbs.mui.ac.ir/article-1-1746-en.html> | No full-text in English. |
| Zhang, Z., Gao, X., Zhou, Y., Yu, C., Pimolsettapun, J., Yang, L., & Zhao, Y. (2020). Study on the combination of brief psychodynamic psychotherapy with Viagra in the treatment of non-organic ED. *General Psychiatry*, *33*(5), e100184. <https://doi.org/10.1136/gpsych-2019-100184> | Not clear if random allocation was used. Author could not be reached. |
| Ziadni, M. S., Carty, J. N., Doherty, H. K., Porcerelli, J. H., Rapport, L. J., Schubiner, H., & Lumley, M. A. (2018). A life-stress, emotional awareness, and expression interview for primary care patients with medically unexplained symptoms: A randomized controlled trial. Health Psychology, 37(3), 282–290. <https://doi.org/10.1037/hea0000566> | Single-session intervention. |

| **Characteristics of the included studies:** | | | | | | | | | | | |
| --- | --- | --- | --- | --- | --- | --- | --- | --- | --- | --- | --- |
| **Study reference** | **Country** | ***n*** | **Primary diagnosis** | **Age**  **group** | **Treatment format** | **EDT model** | **Control condition** | **# of sessions** | **Primary outcome** | **Follow-up (months)** | **RCT-PQRS** |
| Abbasi & Manavipour (2023) | Iran  (non-western) | 36 | Oppositional Defiant Disorder  (mixed/other) | Child /  adolescent | Individual | ISTDP | No treatment (inactive) | 8 | ODDRS | N/A | 12 |
| Abbass et al. (2008) | Canada  (western) | 27 | Personality disorders  (personality) | Adult | Individual | ISTDP | Minimal contact  (inactive) | 27.7 | Combined:  BSI, IIP | N/A | 29 |
| Ajilchi et al. (2016) | Iran  (non-western) | 32 | Major depression  (mood) | Adult | Individual | ISTDP | Waitlist  (inactive) | 15 | BDI | 12 | 26 |
| Bressi et al. (2010) | Italy  (western) | 60 | Mixed diagnoses  (mixed/other) | Adult | Individual | Malan | TAU  (inactive) | 40 | Combined:  GSI, IIP,  CGI | N/A | 29 |
| Brodaty & Andrews (1983) | Australia  (western) | 36 | Mixed diagnoses  (mixed/other) | Adult | Individual | Mixed (Malan + others) | Family GP  (inactive) | 8 | GHQ | 12 | 16 |
| Bögels et al. (2014) | Netherlands  (western) | 47 | Social anxiety disorder  (anxiety) | Adult | Individual | Malan | CBT  (active) | 31.4 | SAC | 12 | 31 |
| Chavooshi et al. (2016a) | Iran  (non-western) | 63 | Medically unexplained pain  (somatic) | Adult | Individual | ISTDP | MBSR  (active)  TAU  (inactive) | 20 | NPRS | 3 | 11 |
| Chavooshi et al. (2016b) | Iran  (non-western) | 100 | Medically unexplained pain  (somatic) | Adult | Individual | ISTDP | TAU  (inactive) | 16 | NPRS | 6 | 30 |
| Chavooshi et al. (2017) | Iran  (non-western) | 341 | Medically unexplained pain  (somatic) | Adult | Individual | ISTDP | CBT  (active) | 11.9 | NPRS | 3 | 32 |
| Dare et al. (2001) | UK  (western) | 84 | Anorexia nervosa  (mixed/other) | Adult | Individual | Malan | Family therapy + CAT  (active)  TAU  (inactive) | 24.9 | Study defined improvement | N/A | 26 |
| Emmelkamp et al. (2006) | Netherlands  (western) | 62 | Avoidant personality disorder  (personality) | Adult | Individual | Mixed (Malan + others) | CBT  (active)  Waitlist  (inactive) | 20 | Combined:  LWASQ, PDBQ, SPAI, AS | N/A | 23 |
| Farzadkia et al. (2023) | Iran  (non-western) | 36 | Fibromyalgia (somatic) | Adult | Individual | ISTDP | MBSR  (active)  Waitlist  (inactive) | 8 | ICAF | 3 | 15 |
| Hellerstein et al. (1998) | USA  (western) | 49 | Personality disorders  (personality) | Adult | Individual | ISTDP | BSP  (active) | 28.5 | Combined:  PTC, GSI, IIP | 6 | 23 |
| Heshmati et al. (2023) | Iran  (non-western) | 86 | Depression (mood) | Adult | Individual | ISTDP | Waitlist  (inactive) | 18.9 | WAI-depression | 3 | 35 |
| Johansson et al. (2013) | Sweden  (western) | 57 | Depression subgroup  (mood) | Adult | Internet | APT (Frederick + McCullough) | Clinical monitoring  (inactive) | N/A | PHQ-9 | N/A | 32 |
| Johansson et al. (2013) | Sweden  (western) | 43 | Anxiety subgroup  (anxiety) | Adult | Internet | APT (Frederick + McCullough) | Clinical monitoring  (inactive) | N/A | GAD-7 | N/A | N/A |
| Johansson et al. (2017) | Sweden  (western) | 72 | Social anxiety disorder  (anxiety) | Adult | Internet | APT (Frederick + McCullough) | Waitlist  (inactive) | N/A | LSAS | N/A | 36 |
| Knekt & Lindfors (2004, 2008) | Finland  (western) | 198 | Mixed diagnoses  (mixed/other) | Adult | Individual | Mixed  (Malan + others) | SFT  (active) | 15.3 | Combined:  BDI, HAM-D, SCL-90-A, HAM-A | 36^a^ | 32 |
| Knijnik et al. (2004) | Brazil  (non-western) | 40 | Social anxiety disorder  (anxiety) | Adult | Group | Malan | Credible placebo control  (inactive) | 12 | LSAS | N/A | 20 |
| Knijnik et al. (2008) | Brazil  (non-western) | 58 | Social anxiety disorder  (anxiety) | Adult | Group + medication | Other  (Malan) | Medication alone  (inactive) | 12 | CGI | N/A | 29 |
| Lindqvist et al. (2020) | Sweden  (western) | 76 | Adolescent depression (mood) | Child /  adolescent | Internet | Mixed  (Malan + others) | Supportive waitlist (inactive) | N/A | QIDS-A17-SR | N/A | 37 |
| Lumley et al. (2017) | USA  (western) | 230 | Fibromyalgia  (somatic) | Adults | Group | EAET | Education group  (inactive)  CBT group  (active) | 8 | BPI | 6 | 35 |
| Maina et al. (2005) | Italy  (western) | 30 | Minor depression  (mood) | Adult | Individual | Malan | BSP  (active)  Waitlist  (inactive) | 19.6 | HAM-D | 6 | 25 |
| Maina et al. (2007) | Italy  (western) | 37 | Major depression  (mood) | Adult | Individual + medication | Malan | BSP + medication  (active) | 15.5 | HAM-D | 6 | 30 |
| Maina et al. (2009) | Italy  (western) | 148 | Major depression  (mood) | Adult | Individual + medication | Malan | Medication alone  (inactive) | 18.3 | HAM-D | 48 | 32 |
| Maina et al. (2010) | Italy  (western) | 57 | OCD + major depression  (anxiety) | Adult | Individual + medication | Malan | Medication alone  (inactive) | 14 | Combined:  HAM-D, Y-BOCS | 6 | 28 |
| Marmar et al. (1988) | USA  (western) | 61 | Unresolved grief  (mixed/other) | Adult | Group | Mixed  (Malan + others) | Self-help group  (inactive) | 12 | GSI | 12 | 20 |
| Maroti et al. (2022) | Sweden  (western) | 106 | Somatic symptom disorder  (somatic) | Adult | Internet | EAET | Waitlist  (inactive) | N/A | PHQ-15 | 4 | 36 |
| Martini et al. (2011) | Italy  (western) | 35 | Panic disorder +  depressive symptoms  (anxiety) | Adult | Individual + medication | Malan | BSP + medication  (active) | 19.9 | Combined:  HAM-A, HAM-D | 6 | 26 |
| Mechler et al. (2022) | Sweden  (western) | 272 | Adolescent depression (mood) | Child /  adolescent | Internet | Mixed  (Malan + others) | ICBT  (active) | N/A | QIDS-A17-SR | N/A | 38 |
| Mechler et al. (2024) | Sweden  (western) | 181 | Social anxiety disorder  (anxiety) | Adult | Internet | Mixed  (Malan + others) | Waitlist  (inactive) | N/A | LSAS | N/A | 35 |
| Milo et al. (2023) | Italy  (western) | 60 | Inflammatory bowel disease  (somatic) | Child /  adolescent | Individual + medication | Malan | Medication alone  (inactive) | 8 | Study defined remission maintenance at follow-up | 12 | 33 |
| Mowlaie et al. (2018) | Iran  (non-western) | 30 | Adult separation anxiety disorder  (anxiety) | Adult | Individual | APT (McCulllough) | BEP  (active) | 12 | DASS-A | 2 | 12 |
| Nader-Mohammadi Moghadam et al. (2015) | Iran  (non-western) | 38 | Social anxiety disorder  (anxiety) | Adult | Individual | APT  (McCulllough) | Medication  (active)  Waitlist  (inactive) | 21 | SPIN | N/A | 18 |
| Nakhaei Moghadam et al. (2024) | Iran  (non-western) | 30 | Chronic pain (somatic) | Adult | Individual | ISTDP | No treatment (inactive) | 15.0 | SQ | 2 | 13 |
| Orvati Aziz et al. (2020) | Iran  (non-western) | 36 | Generalized anxiety disorder  (anxiety) | Adult | Individual | ISTDP + CBT  (other) | CBT  (active)  No treatment  (inactive) | 15.0 | HRSA | N/A | 12 |
| Pierloot & Vink (1978) | Belgium  (western) | 22 | Mixed anxiety  (anxiety) | Adult | Individual | Malan | Behavior therapy  (active) | 19.7 | TMAS | 3 | 11 |
| Piper et al. (1990) | Canada  (western) | 144 | Mixed diagnoses  (mixed/other) | Adult | Individual | Mixed  (Malan + others) | Waitlist  (inactive) | 18.6 | GSI | N/A | 23 |
| Prout et al. (2022) | USA  (western) | 43 | Oppositional defiant disorder (mixed/other) | Child / adolescent | Individual | Mixed  (McCullough, Malan) | Waitlist  (inactive) | 16.0 | ODDRS | N/A | 34 |
| Rahmani et al. (2011) | Iran  (non-western) | 50 | Major depression  (mood) | Adult | Individual | ISTDP | Medication  (active) | 15.0 | BDI | N/A | 14 |
| Rahmani et al. (2020a; 2020b) | Iran  (non-western) | 70 | Social anxiety disorder  (anxiety) | Adult | Individual | ISTDP | Waitlist  (inactive) | 10.0 | LSAS | 6 | 17 |
| Reneses et al. (2013) | Spain  (western) | 42 | Borderline personality disorder  (personality) | Adult | Individual | Mixed  (Trujillo, Davanloo) | TAU  (inactive) | 20 | Combined:  GSI, BIS, SASS | N/A | 24 |
| Rosso et al. (2012) | Italy  (western) | 88 | Mild/moderate depression  (mood) | Adult | Individual | Malan | BSP  (active) | 18.6 | HAM-D | 6 | 29 |
| Rostami Ravari et al. (2024) | Iran  (non-western) | 16 | Functional gastrointestinal disorders (somatic) | Adult | Individual | ISTDP | No treatment (inactive) | 15.0 | GSRS | 2 | 11 |
| Salminen et al. (2008) | Finland  (western) | 51 | Major depression  (mood) | Adult | Individual | Mixed (Malan + Mann) | Medication  (active) | 16 | Combined: HAM-D, BDI | N/A | 28 |
| Shafiei et al. (2024) | Iran  (non-western) | 30 | Irritable bowel disease  (somatic) | Adult | Individual | ISTDP | Waitlist  (inactive) | 16 | Combined:  Severity and frequency of IBS symptoms | 2.5 | 14 |
| Shahverdi et al. (2024) | Iran  (non-western) | 30 | Tension type headache  (somatic) | Adult | Individual | ISTDP | Waitlist  (inactive) | 16 | NPRS | 2.5 | 14 |
| Svartberg et al. (2004) | Norway  (western) | 50 | Cluster-C personality disorders  (personality) | Adult | Individual | APT (McCullough) | Cognitive therapy  (active) | 40 | GSI | 24 | 34 |
| Tasca et al. (2006) | Canada  (western) | 135 | Binge eating disorder  (mixed/other) | Adult | Group | Mixed  (McCullough, Malan) | CBT group  (active)  Waitlist  (inactive) | 12.4 | Days binged past week | 6 | 37 |
| Thakur et al. (2016) | USA  (western) | 106 | Irritable bowel syndrome  (somatic) | Adult | Individual | EAET | Relaxation training  (active)  Waitlist  (inactive) | 3 | IBS Symptom Severity Scale | 3 | 23 |
| Town et al. (2017, 2020) | Canada  (western) | 60 | Treatment resistant depression (mood) | Adult | Individual | ISTDP | TAU  (inactive) | 16.1 | HAM-D | 18^a^ | 44 |
| Town et al. (2024) | Canada  (western) | 37 | Somatic Symptom Disorder (somatic) | Adult | Individual | ISTDP | Waitlist  (inactive) | 8.6 | SOMS-7 | 6 | 36 |
| Trowell et al. (2007) | UK, Greece, Finland  (western) | 72 | Childhood depression (mood) | Child / adolescent | Individual | Mixed  (Malan + Davanloo) | Family therapy (active) | 24.7 | Kiddie-SADS | 6 | 23 |
| Wiborg & Dahl (1996) | Norway  (western) | 40 | Panic disorder  (anxiety) | Adult | Individual + medication | Mixed  (Malan, Davanloo + others) | Medication alone  (inactive) | 15 | PAAS | 18 | 27 |
| Winston et al. (1994) | USA  (western) | 81 | Personality disorders  (personality) | Adult | Individual | Mixed  (Malan, Davanloo + others) | BAP  (active)  Waitlist  (inactive) | 40.3 | GSI | N/A | 22 |
| Yarns et al. (2020) | USA  (western) | 53 | Chronic pain (somatic) | Adult | Group | EAET | CBT group  (active) | 9 | BPI | 3 | 31 |
| Yarns et al. (2024) | USA  (western) | 126 | Chronic pain (somatic) | Adult | Group | EAET | CBT  (active) | 9 | BPI | 6 | 33 |
| Ziapour et al. (2023) | Iran  (non-western) | 60 | Depression  (mood) | Adult | Individual | ISTDP | Waitlist  (inactive) | 20 | BDI | N/A | 12 |
| *Note.* APT = Affect Phobia Therapy; AS = Avoidance scale; BAP = Brief Adaptive Psychotherapy; BDI = Beck Depression Inventory; BIS = Barratt Impulsivity Scale; BSI = Brief Symptom Inventory; BSP = Brief Supportive Therapy; CAT = Cognitive Analytic Therapy; CBT= Cognitive-Behavioral Therapy; CGI = Clinical Global Impression scale; DASS-A = Depression, anxiety, and stress scale-21, anxiety subscale; EDT = Experiential Dynamic Therapy; GAD-7 = Generalized Anxiety Disorder scale; GHQ = Global Health Questionnaire; GSI = Global Symptom Index of the Symptom Check List-90; GP = general practitioner; GSRS = Gastrointestinal Symptom Rating Scale; HAM-A = Hamilton Anxiety Rating Scale; HAM-D = Hamilton Depression Rating Scale; HRSA = Hamilton Anxiety Rating Scale; IBS = Irritable bowel syndrome; ICAF = Combined Index of Severity of Fibromyalgia; IIP = Inventory of Interpersonal Problems; ISTDP = Intensive Short-Term Dynamic Psychotherapy; ITT = Intent-to-treat; Kiddie-SADS = Schedule for Affective Disorders and Schizophrenia for school-age children; LSAS = Liebowitz Social Anxiety Scale; LWASQ = Lehrer Woolfolk Anxiety Symptoms Questionnaire; MBSR = Mindfulness-based stress reduction; NPRS = Numerical pain rating scale; ODDRS = Oppositional Defiant Disorder Rating Scale; PAAS = Panic Attack and Anxiety Scale; PDBQ = Personality Disorder Belief Questionnaire; PHQ-9 = Patient Health Questionnaire Depression Scale; PHQ-15 = Patient Health Questionnaire Somatic Symptoms; PPC = Psychopathology Composite; PTC = Patient-rated target complaint; QIDS-A17-SR = Quick Inventory of Depressive Symptomatology for Adolescents; RCT-PQRS = Randomized Controlled Trial of Psychotherapy Quality Rating Scale (total score); SAC = Social Anxiety Composite; SASS = Social Adjustment Self-evaluation Scale; SCL-90-A = Symptom Check List Anxiety subscale; SFT = Solution-Focused Therapy; SPAI = Social Phobia Anxiety Inventory; SOMS-7 = Somatic symptoms severity score; SPIN = Social Phobia Inventory; SQ = Somatization questionnaire; TAU = Treatment-as-usual; TMAS = Taylor Manifest Anxiety Scale; UK = United Kingdom; WAI-Depression = Weinberger Adjustment Inventory–depression subscale; Y-BOCS = Yale-Brown Obsessive Compulsive Scale  ^a^ = Follow-up data extracted from secondary publication | | | | | | | | | | | |

**Forest and funnel plots for EDTs versus inactive controls at post-treatment**


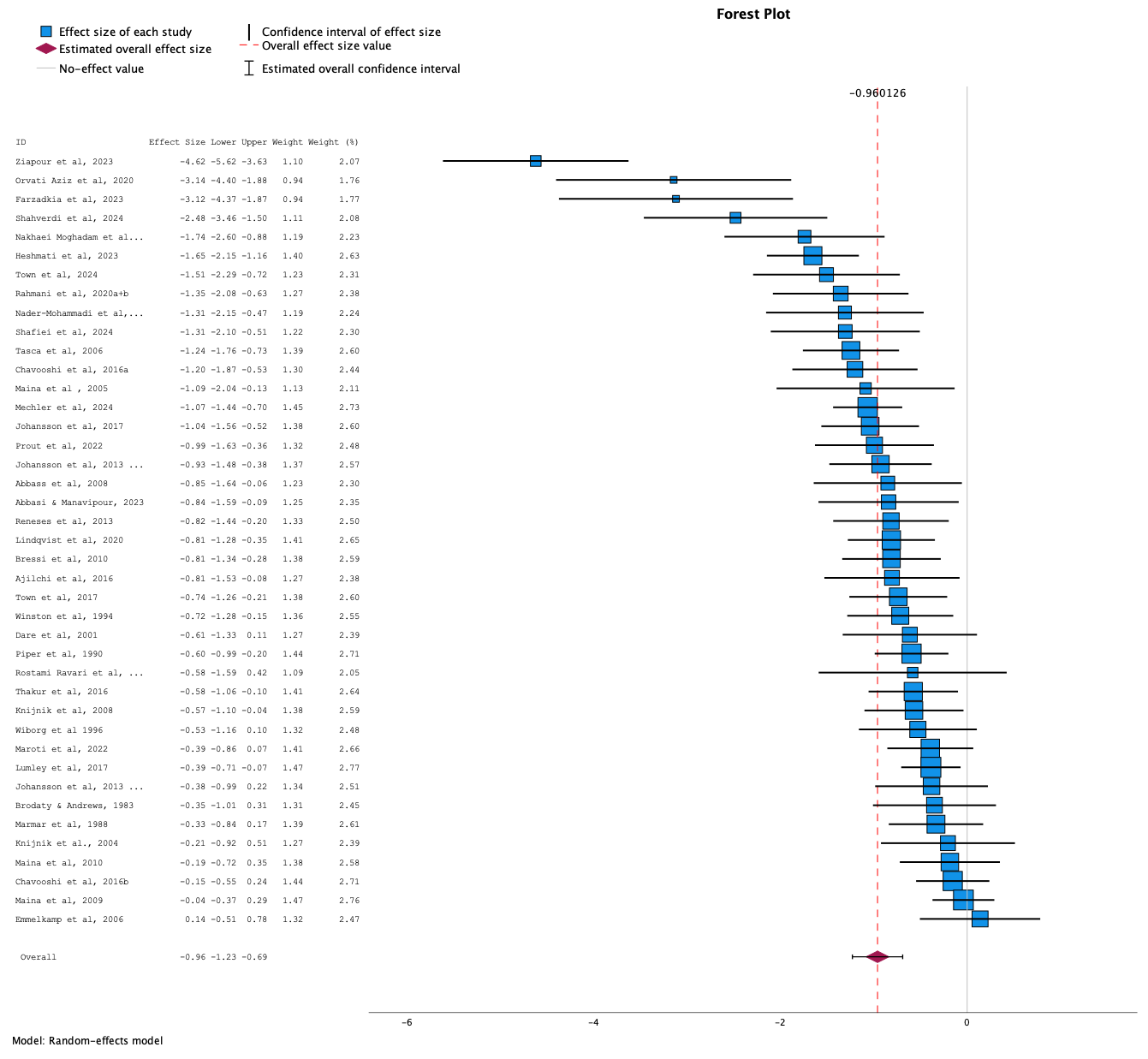


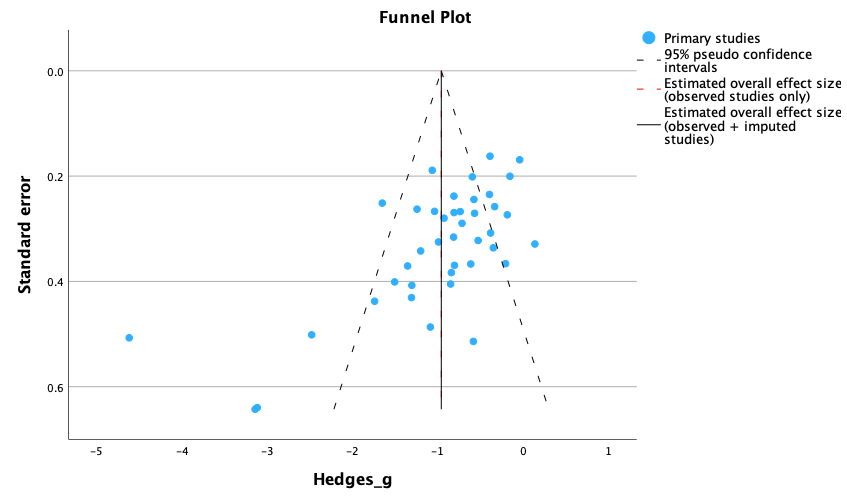


**Forest and funnel plots for EDTs versus inactive controls at follow-up**


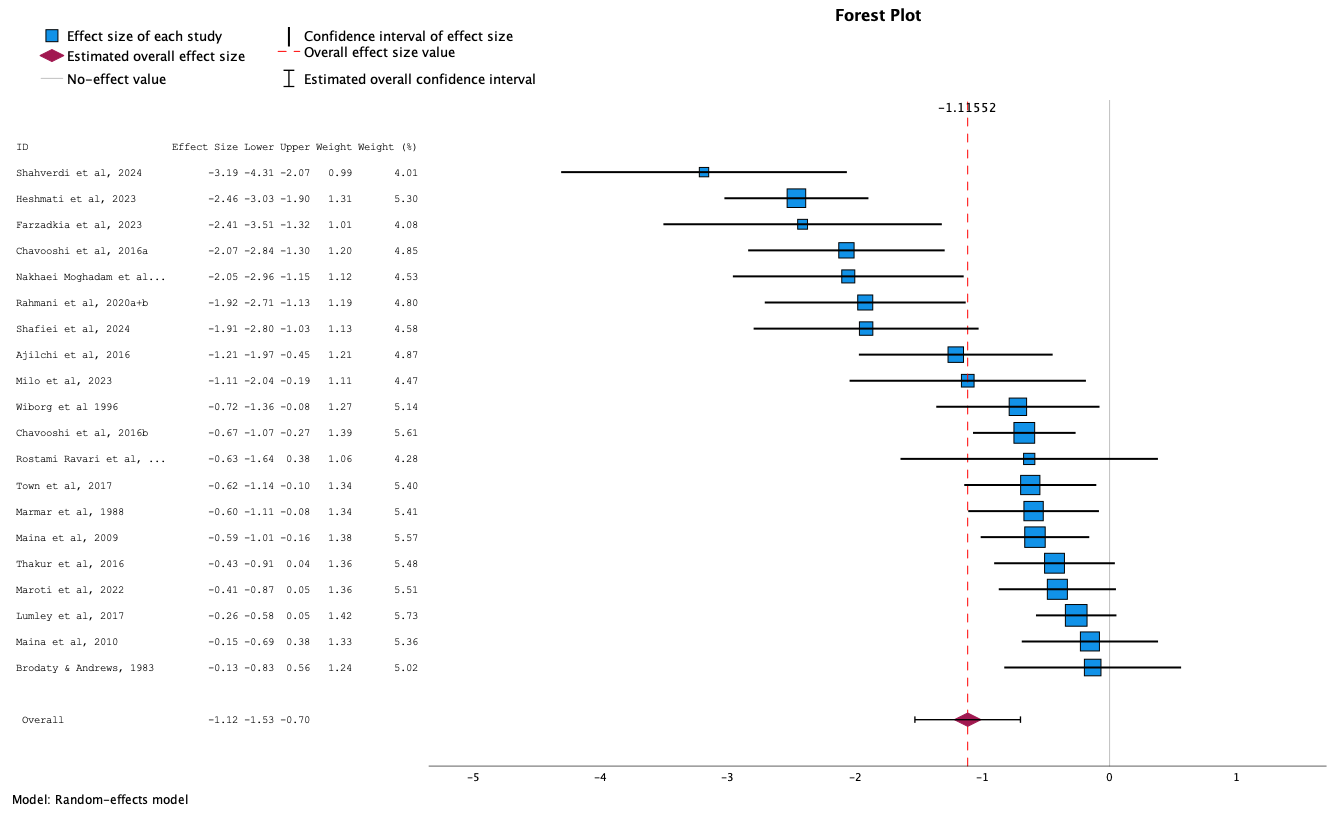


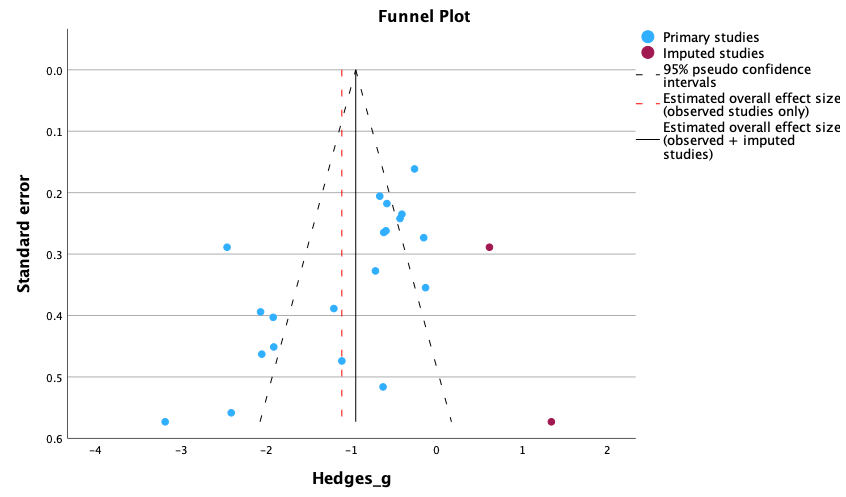


**Forest and funnel plots for EDTs versus active controls at post-treatment**


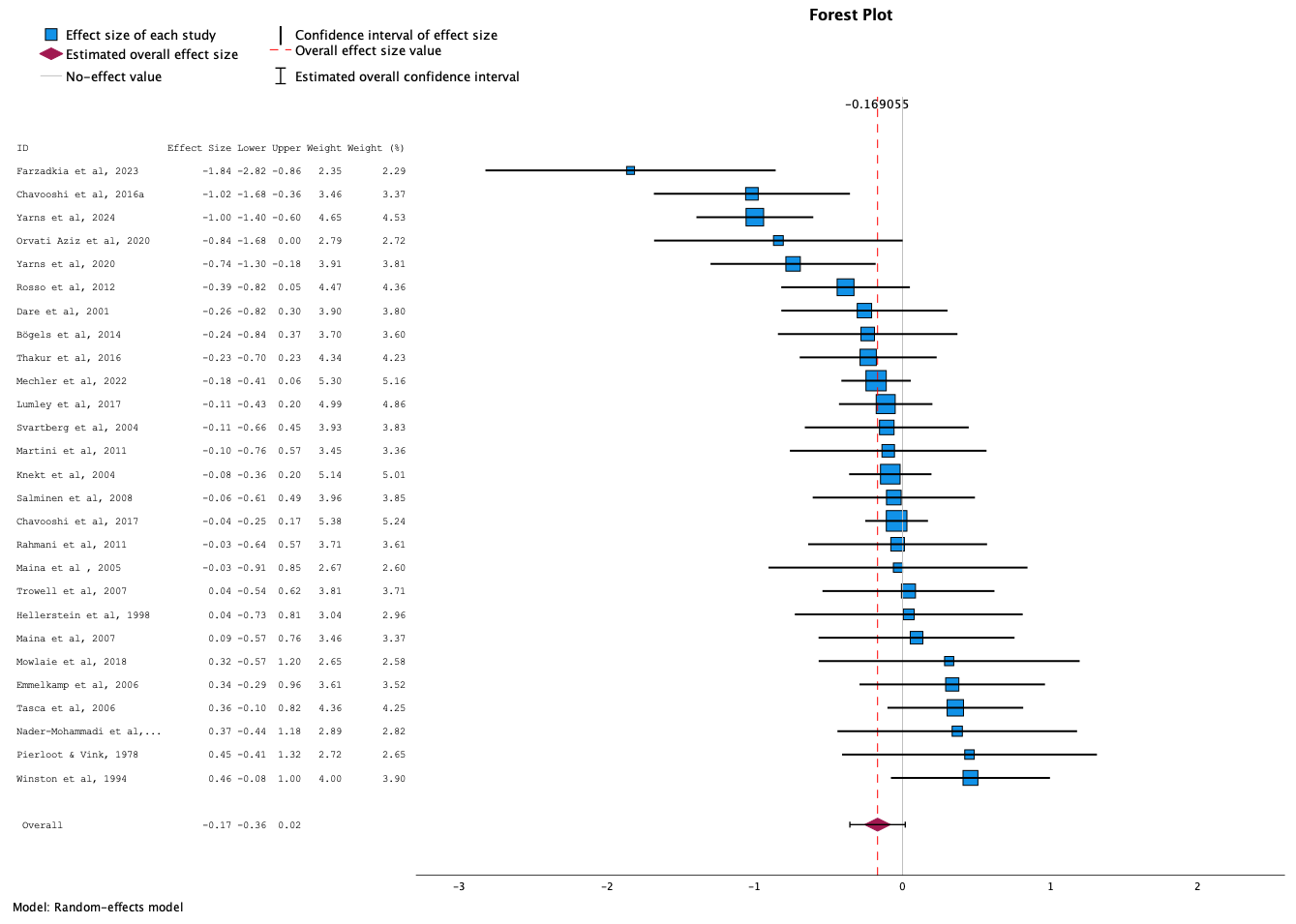


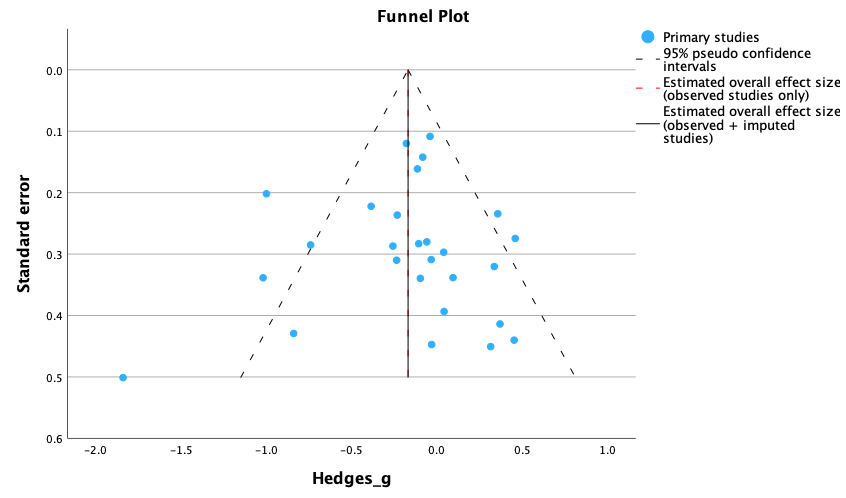


**Forest and funnel plots for EDTs versus active controls at follow-up**


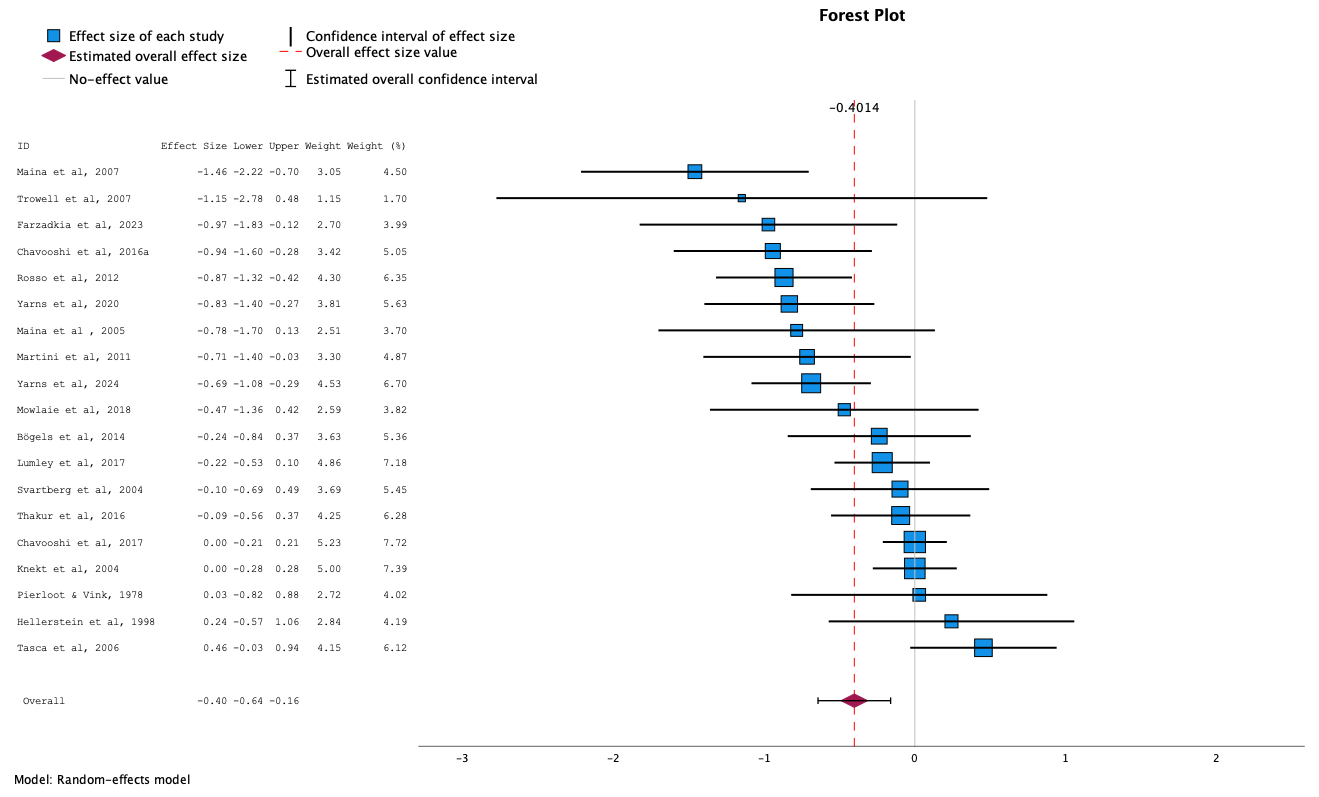


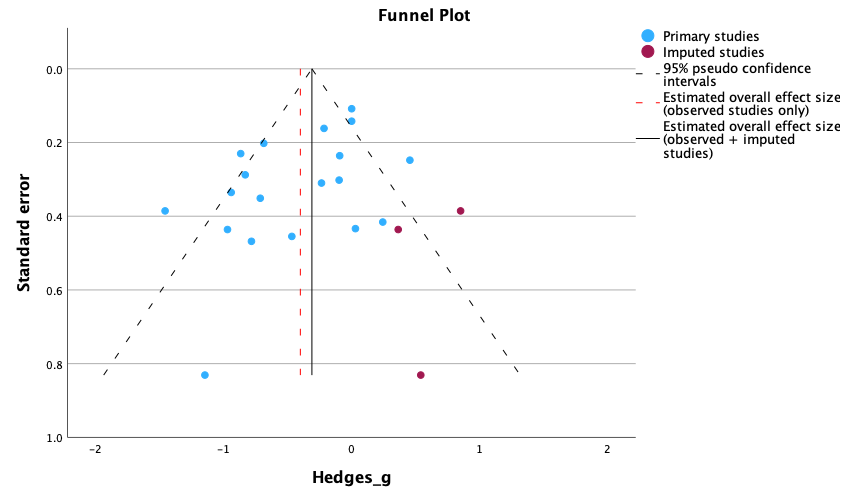


**Subgroup forest-plot and funnel-plots for analyses of EDTs versus active controls at follow-up**


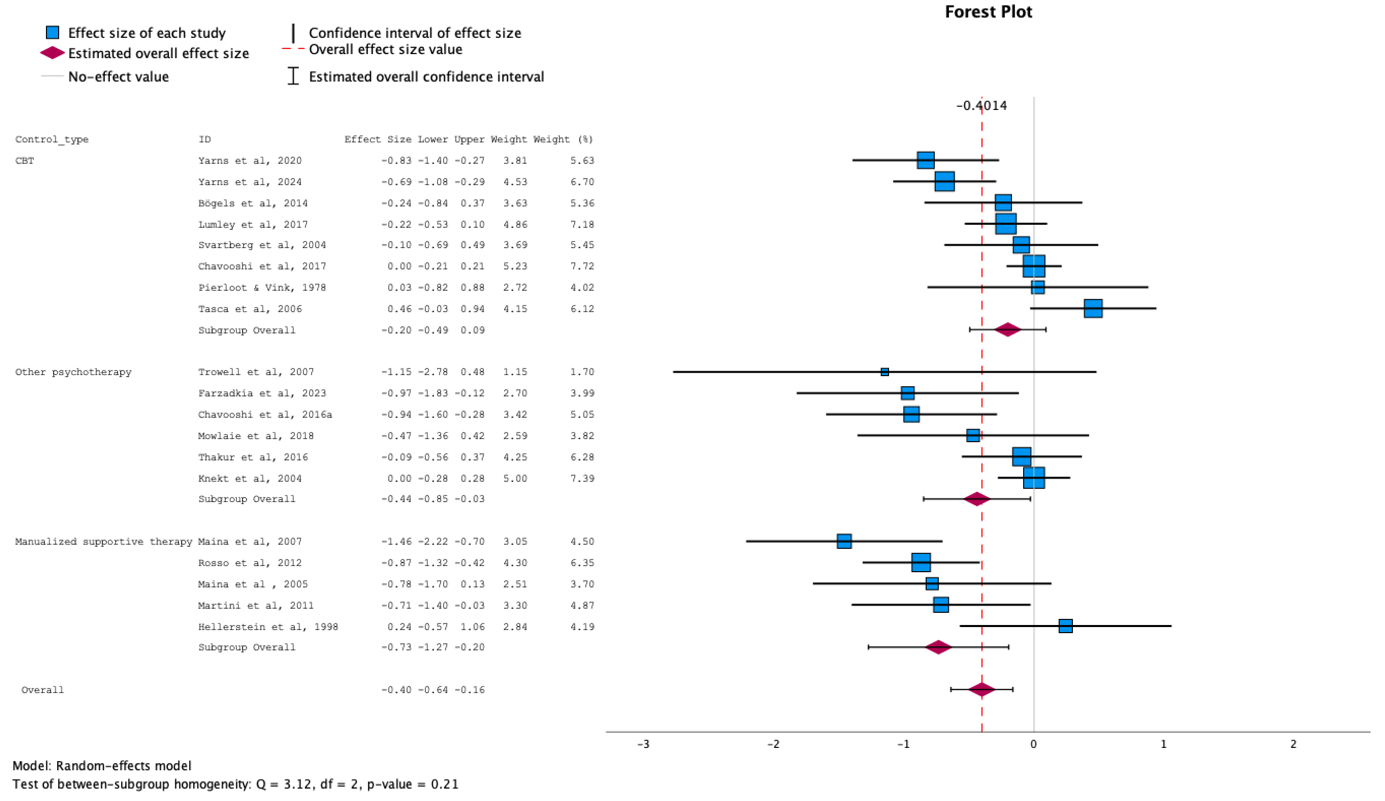


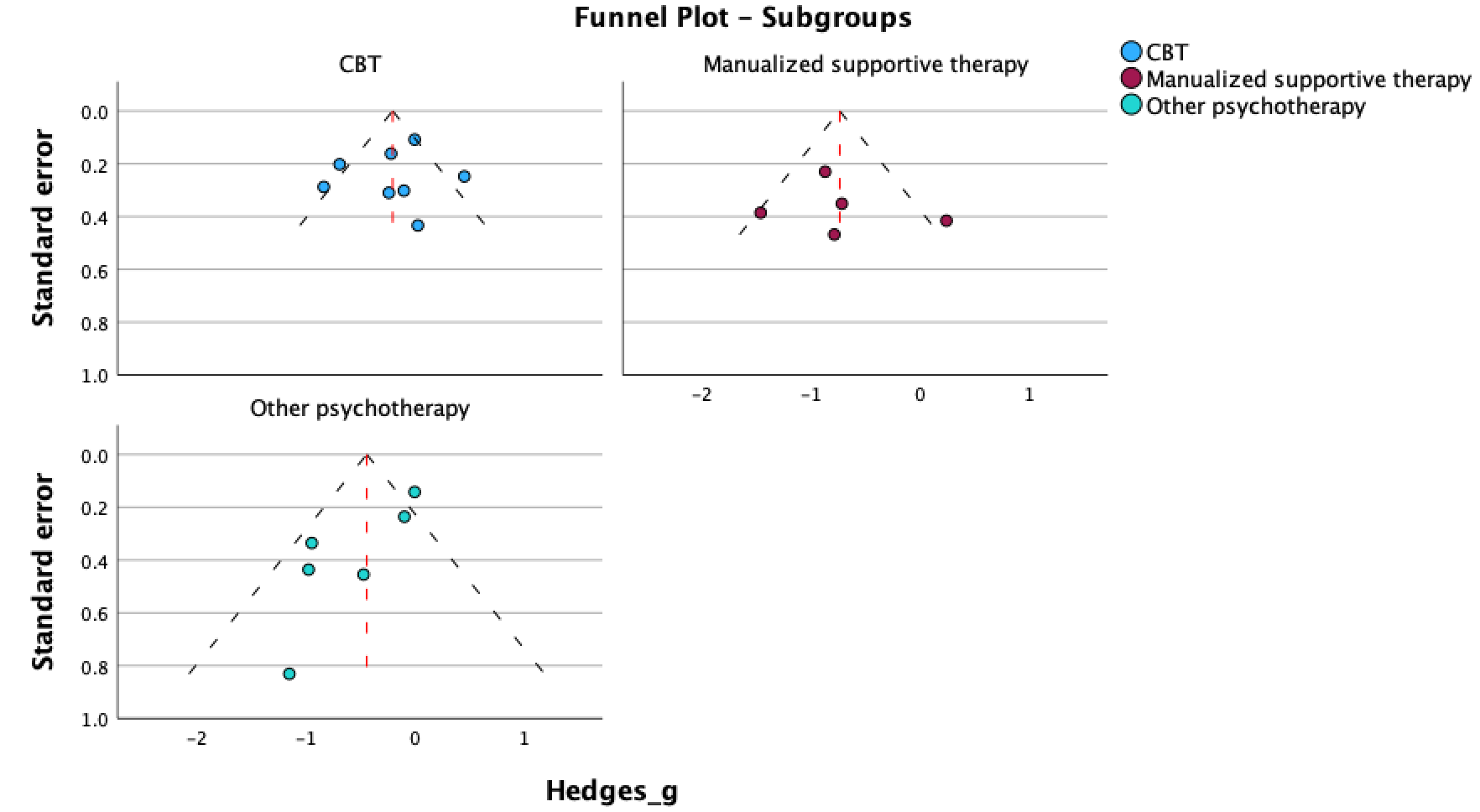


**Bubble-plots for the relationship between study quality and effects of EDTs versus inactive controls at post-treatment and follow-up**

**
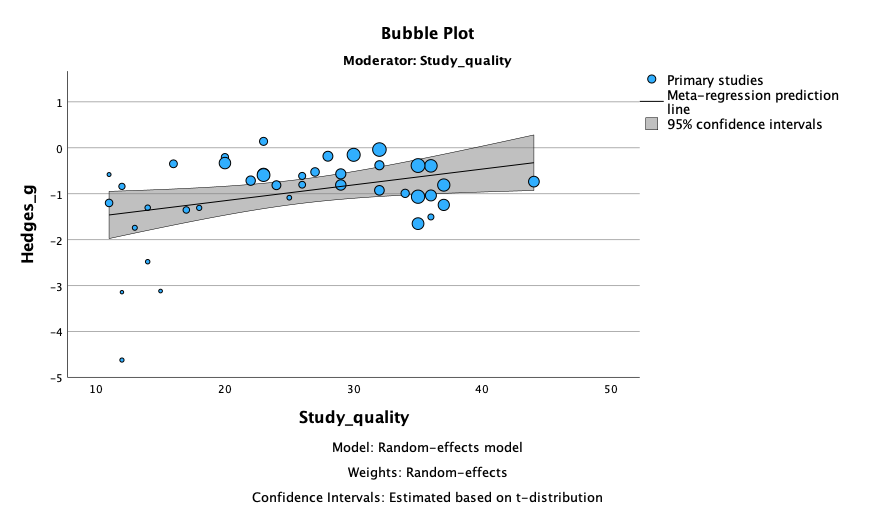
**

**
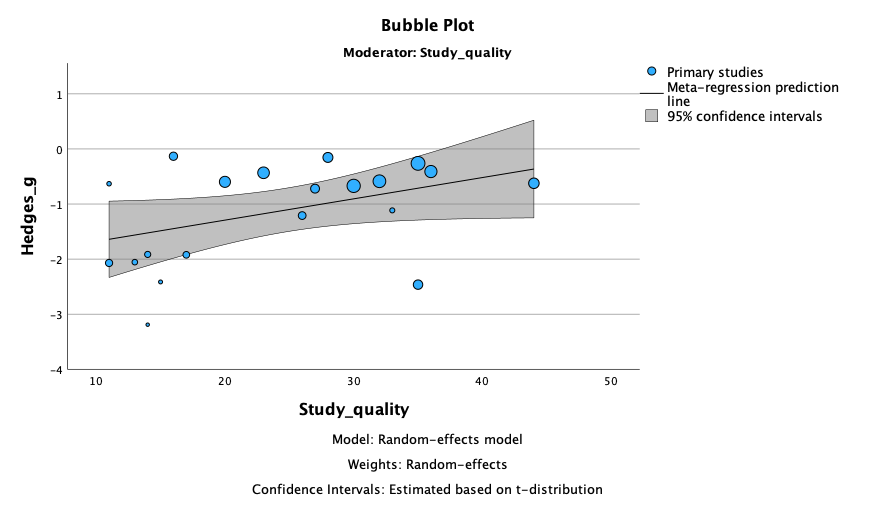
**

**Bubble-plots for the relationship between study quality and effects of EDTs versus active controls at post-treatment and follow-up**

**
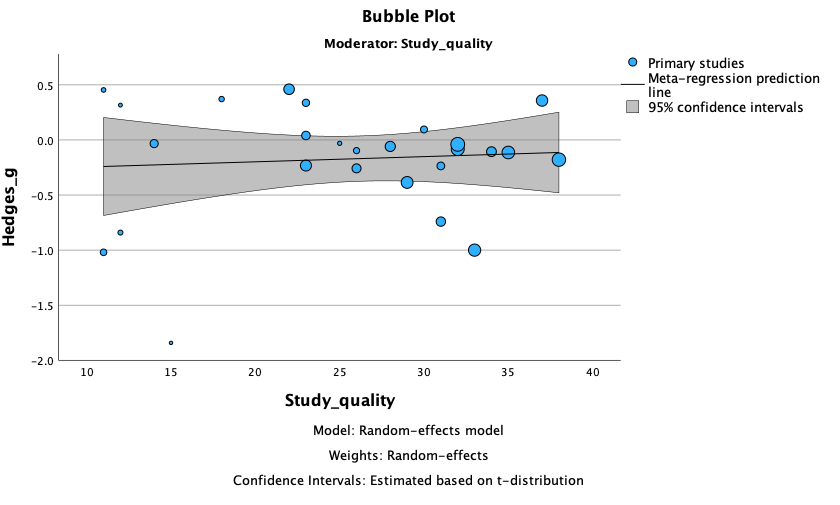
**

**
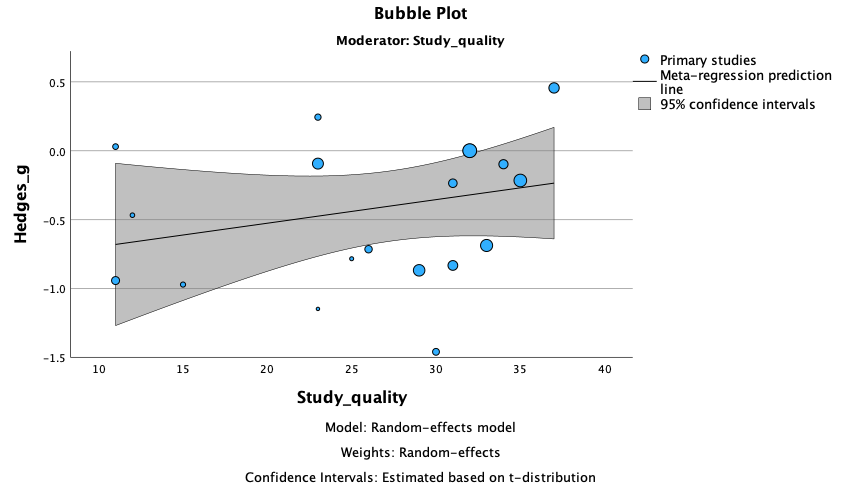
**
